# A Methodological Approach Using rAAV Vectors Encoding Nanobody-Based Biologics to Evaluate ARTC2.2 and P2X7 *In Vivo*

**DOI:** 10.3389/fimmu.2021.704408

**Published:** 2021-08-19

**Authors:** Henri Gondé, Mélanie Demeules, Romain Hardet, Allan Scarpitta, Marten Junge, Carolina Pinto-Espinoza, Rémi Varin, Friedrich Koch-Nolte, Olivier Boyer, Sahil Adriouch

**Affiliations:** ^1^Normandie University, UNIROUEN, INSERM U1234, Pathophysiology, Autoimmunity, Neuromuscular Diseases and Regenerative THERapies, Rouen, France; ^2^Rouen University Hospital, Department of Pharmacy, Rouen, France; ^3^Institute of Immunology, University Medical Center Hamburg-Eppendorf, Hamburg, Germany; ^4^Rouen University Hospital, Department of Immunology and Biotherapy, Rouen, France

**Keywords:** P2X7 (purino) receptor, AAV vectors, nanobodies (V_HH_), animal models, extracellular ATP (eATP), extracellular NAD^+^, methodological approach

## Abstract

On murine T cells, mono-ADP ribosyltransferase ARTC2.2 catalyzes ADP-ribosylation of various surface proteins when nicotinamide adenine dinucleotide (NAD^+^) is released into the extracellular compartment. Covalent ADP-ribosylation of the P2X7 receptor by ARTC2.2 thereby represents an additional mechanism of activation, complementary to its triggering by extracellular ATP. P2X7 is a multifaceted receptor that may represents a potential target in inflammatory, and neurodegenerative diseases, as well as in cancer. We present herein an experimental approach using intramuscular injection of recombinant AAV vectors (rAAV) encoding nanobody-based biologics targeting ARTC2.2 or P2X7. We demonstrate the ability of these *in vivo* generated biologics to potently and durably block P2X7 or ARTC2.2 activities *in vivo*, or in contrast, to potentiate NAD^+^- or ATP-induced activation of P2X7. We additionally demonstrate the ability of rAAV-encoded functional heavy chain antibodies to elicit long-term depletion of T cells expressing high levels of ARTC2.2 or P2X7. Our approach of using rAAV to generate functional nanobody-based biologics *in vivo* appears promising to evaluate the role of ARTC2.2 and P2X7 in murine acute as well as chronic disease models.

## Introduction

Nicotinamide adenine dinucleotide (NAD^+^) is a key molecule in cellular metabolism and acts as an intermediate in several essential enzymatic reactions (1). In addition, in response to cellular stress, intracellular NAD^+^ is released into the extracellular compartment and serves as a substrate for various ectoenzymes (2, 3). Mono-ADP ribosyl transferases (ART) represent a family of ectoenzymes that use extracellular NAD^+^ to catalyze posttranslational modification of cell surface proteins by the transfer of ADP-ribose to specific amino-acid residues (4, 5). In mice, the ART family comprises six members: ARTC1-5, including two isoforms of ARTC2, termed ARTC2.1 and ARTC2.2 (6). While ARTC2.1 is enzymatically inactive in the absence of reducing agents, ARTC2.2 is active in standard conditions and is able to ADP-ribosylate multiple cell-surface protein-targets when NAD^+^ is present in the extracellular space (7, 8). ARTC2.2 is localized predominantly on the surface of murine T cells as a 35 kDa GPI-anchored ectoenzyme. Although its levels of expression varies depending on mouse strain and cell-activation status, membrane expression of ARTC2 remains overall higher on CD8^+^ T cells as compared to CD4^+^ T cells (9). When murine T cells are exposed to micromolar levels of extracellular NAD^+^, ARTC2.2 catalyzes the ADP-ribosylation of exposed arginines in several cell surface protein targets, including the purinergic P2X7, a well described protein expressed by immune cells and involved in immune regulation (10, 11).

P2X7 assembles at the cell surface as a homo-trimeric receptor that forms a nonselective ion channel upon gating with high extracellular ATP concentrations (*i.e.*, in the hundreds micromolar range). Depending on ATP concentration and on the extent of cell exposition, activation of P2X7 receptor can lead to multiple cellular events starting by the rapid activation of surface metalloproteases (leading to shedding for instance of CD62L and CD27) and by the externalization of phosphatidylserine (11). Prolonged P2X7 receptor activation induces the formation of nonselective pores and to massive membrane depolarization, ultimately leading to cell death (3). Interestingly, prolonged P2X7 activation can also be triggered by brief exposition to extracellular NAD^+^. Indeed, in the presence of extracellular NAD^+^, ARTC2.2 catalyzes covalent ADP-ribosylation of P2X7 at the arginine residue 125, located in the vicinity of the ATP-binding site, and thereby triggers the activation of P2X7 receptor (10). Remarkably, much lower concentrations of extracellular NAD^+^ (*i.e.*, in the micromolar range) are sufficient to activate P2X7 receptor and to induce cell death (12, 13). This process was termed NAD-induced cell death (NICD) and demonstrated to play a major role *in vivo* in the fate and regulation of immune cells that express high levels of ARTC2.2 and P2X7, including regulatory T cells (Treg), invariant NKT cells, follicular helper T cells (Tfh), and tissue-resident memory T cells (T_RM_) (12, 14–18).

Nanobodies are derived from unconventional natural antibodies devoid of light chains that are found in llamas and other camelids (19–21). The single-chain variable fragment of the so-called heavy-chain antibodies is termed V_HH_ or nanobody. Nanobodies exhibit similar specificities and affinities than conventional antibodies but are smaller in size (15 kDa) and present a complementary determining region 3 (CDR3) that is usually longer, with the remarkable propensity to reach protein cavities that are otherwise difficult to target with conventional antibodies, offering opportunities to engineer these molecules into original biologics (22). Such cavities often correspond to functional regions and allosteric sites, conferring to nanobodies the ability to act as modulators of enzyme and receptor activities (*e.g.*, potentiating or blocking). Anti-ARTC2.2 nanobodies have been isolated by phage display from llamas immunized with cDNA expression vectors encoding full-length ARTC2.2 (23). Nanobodies s-14, s+16a, s+16b and l-17 are able to bind with high specificity cell line stably transfected to express ARTC2.2. In addition, T cells can be protected from NICD by ARTC2.2-blocking nanobodies s+16a, s+16b and l-17, but not s-14 (23). In additional studies, intravenous (i.v.) injection of the ARTC2.2-blocking nanobody s+16a prevented NICD in cells co-expressing ARTC2.2 and P2X7 *ex vivo* as well as *in vivo*. ARTC2.2 antagonism has hence been recognized as a crucial step for the functional studies of Treg, NKT and T_RM_ subsets (14–16, 24). Anti-P2X7 nanobodies were generated in a similar manner (25, 26). Taking advantage of the single-chain structure of nanobodies, we derived highly specific P2X7-potentiating nanobody 14D5 and P2X7-blocking nanobody 13A7 either as homodimers fused to the Alb8 albumin-binding nanobody (*i.e.*, to provide improved half-life *in vivo*) or fused to the Fc-region of mouse conventional antibody to reconstitute the bivalent heavy chain antibody (hcAb) format of camelids (25, 27). Nanobody-based hcAb therefore offer the possibility not only to increase their half-life *in vivo* but also to provide effector functions related to the Fc-region that can bind to Fc-receptors (FcR) at the surface of immune cells or activate the classical complement cascade (26). Depending on the isotype, it is then possible to promote antibody-dependent cell cytotoxicity (ADCC), complement-dependent cell cytotoxicity (CDC) and antibody-dependent phagocytosis (ADCP). Interestingly, hinge and/or Fc-region engineering can further enhance Fc-fused nanobody half-life or fine tune effector properties by enhancing or abolishing FcγR and/or complement related effector functions (28).

In this study, we report two different strategies for manipulating the ARTC2.2/P2X7 pathway *in vivo*, with the aim of better addressing the versatile role of NAD^+^ and ATP in immune cells. For that, we developed a methodology that rely on nanobody-based biologics expressed directly *in vivo* upon a single intramuscular (i.m.) administration of recombinant AAV vectors (rAAV) encoding the biological constructs. We demonstrated here the ability to durably block the activity of ARTC2.2 enzyme or of the P2X7 ion channel *in vivo* upon a single i.m. injection of the rAAV encoding a construct containing the ARTC2.2-blocking nanobody s+16a, or a construct containing the P2X7-blocking nanobody 13A7. In addition, we provide evidence that P2X7 can be potentiated *in vivo* using an rAAV encoding a construct containing the P2X7-potentiating nanobody 14D5. In another strategy, based on the fusion of specific nanobodies to mouse IgG2a, to generate a heavy chain antibody (hcAb) format, we demonstrate durable depletion of cells expressing high levels of ARTC2.2 *in vivo*. These methodologies represent valuable tools that might be used in future studies to better delineate the role of the NAD^+^/ARTC2.2/P2X7 pathway *in vivo* in various pathophysiological situations including inflammatory diseases, and immune responses to infectious pathogens or to tumor cells.

## Material and Methods

### Mice, Reagents, Antibodies

C57BL/6 wild-type mice obtained from Janvier Labs were used for all experiments. Mice were housed in a specific pathogen-free facility and were aged of 8 weeks at the beginning of experiments. All animal experiments were approved by the local institutional ethic committee.

Adenosine 5’-tri-phosphate disodium salt (A2383) and β-nicotinamide adenine dinucleotide hydrate (N7004) were purchased from Sigma Aldrich. Red blood cell (RBC) lysis/fixation Solution, True-Nuclear transcription factor buffer set, fluorochrome-conjugated streptavidin, and antibodies to CD45 (clone 30-F11), CD4 (RM4-5), CD8 (53-6.7), CD25 (PC-61), CD19 (1D3/CD19), B220 (RA3-6B2), FoxP3 (MF-14), CD27 (LG.3A10), CD62L (MEL-14), CD69 (H1.2F3) or P2X7R (1F11), and purified CD16/CD32 (TruStain FcX) were obtained from Biolegend or Sony Biotechnology. Rabbit polyclonal antibody K1G, specific to mouse P2X7, was described in our previous studies (12, 13). K1G was used to stain P2X7 at the surface of blood myeloid cells as illustrated in **Supplemental Figure 3**, using a secondary donkey anti-rabbit IgG from Jackson ImmunoResearch. Biotinylated polyclonal antibody specific to mouse IgGa was obtained from Jackson ImmunoResearch and monoclonal antibody to ARTC2.2 (Nika102) from Novus Biologicals.

### Flow Cytometry Analyses

For evaluation of P2X7 and ARTC2.2 expression on T cells, splenocytes were collected and single-cell suspensions were prepared and washed using standard procedures. Cells were stained with fluorochrome-conjugated antibodies, including anti-P2X7 and anti-ARTC2.2 or related isotype controls before fixation and red blood cell lysis using the RBC lysis/fixation Solution (Sony biotechnology).

For evaluation of P2X7-dependent shedding of CD27 and CD62L upon *ex vivo* exposition to NAD^+^ or ATP, blood samples were collected, washed, resuspended into PBS (without Ca^2+^ and Mg^2+^), and divided into 4 tubes. Cells were then treated with 30 µM ATP, 150 µM ATP, or 30 µM NAD^+^, or left untreated. After incubation for 15 min at 37°C, cells were washed in cold D-PBS containing 10% FBS, and stained on ice with fluorochrome-conjugated antibodies before fixation and RBC lysis. The percentages of cells co-expressing CD27 and CD62L were then evaluated by flow cytometry.

For evaluation of the binding of the dimHLE constructs on liver NKT cells and liver T_RM_, single cells suspension were prepared and stained with a mouse IgG1 mAb that specifically recognizes the Alb8 nanobody (kindly provided by Dr. Catelijne Stortelers, Ablynx nv, Zwijnarde, Belgium), followed by a secondary mouse IgG1-specific mAb. To evaluate the levels of occupation of the target protein, cells were incubated *in vitro* with a saturating concentration of the same recombinant nanobody construct as the one produced *in vivo*, followed by the same secondary detection reagents. This allowed estimation of signal saturation that correlates with target occupation by bounded biologics. Similar MFI were obtained with or without addition *in vitro* of the corresponding recombinant nanobody construct, confirming that target occupancy reached saturation *in vivo* (data not shown). To distinguish vascular T cells from liver parenchyma cells, a fluorochrome-conjugated CD45-specific mAb has been injected i.v. (CD45iv) 3 min before sacrifice and preparation of liver lymphocytes. This procedure allowed the staining of vascular T cells *in vivo* (CD45iv^high^) while liver resident T cells remained mostly unstained (CD45iv^low^). Untransduced mice additionally received 50 µg of recombinant s+16a-dim construct (i.e., a dimer of s+16a that does not contain the Alb8 nanobody, and that cannot be detected by the anti-Alb8 mAb77 detection system) 30 min before organ collection to prevent NICD and cell loss *ex vivo* during cell preparation, as described earlier (15, 18). All cells were counterstained with mAbs directed against CD45 (coupled to a different fluorochrome than CD45iv), CD4, and CD69 (marker of tissue resident lymphocytes). To distinguish the NKT subset, cells were stained with PE-conjugated CD1d-tetramer loaded with, an analogue of α-galatosylceramide (αGal/Cer), kindly provided by the NIH tetramer core facility.

For evaluation of the binding capacity of the nanobody-based hcAb on T cells surface, samples were collected, washed, resuspended into FACS buffer and stained with isotype specific secondary antibodies, i.e. Ab directed against mouse IgG1 (to detect 13A7-IgG1^LSF^ hcAb), or Ab directed against mouse IgG2a (for detection of 7E2-IgG2a or s-14-IgG2a hcAb). Cells were washed and counterstained with fluorochrome-conjugated antibodies directed against the indicated cell surface markers.

Flow cytometry measurements were performed using a LSRFortessa or a FACSCanto-I (BD Biosciences) apparatus and subsequent analyses were performed using FlowJo software (Tree Star, Ashland, OR).

### Nanobody Constructs

Some of the constructs used in this study were based on nanobody dimers (“dim” format) fused to the Alb8 anti-albumin nanobody (half-life extended “HLE” format) (25, 29). 14D5-dimHLE and s+16a-dimHLE were constructed by fusing the coding sequences of two 14D5 nanobodies or two s+16a nanobodies using a 35-GS linker (GGGGS)_7_. These homodimeric bivalent constructs were then fused to the anti-albumin nanobody Alb8 *via* a 9-GS linker (GGGGSGGGS) (25). The P2X7-blocking 13A7-IgG1^LSF^ hcAb was constructed by fusing the nanobody 13A7 to the hinge region and Fc region of a mutated mouse IgG1 antibody carrying the “LSF” mutations (T252L, T254S, T256F) (30). For the design of the depleting hcAb constructs 7E2-IgG2a and s-14-IgG2a, anti-P2X7 nanobody 7E2 or anti-ARTC2.2 nanobody s-14, were fused to the hinge and Fc-region of a mouse IgG2a using 5-GS linker (GGGGS).

### Production of rAAV and Muscle Transduction

For the production of rAAV, all constructs were cloned into a pFB plasmid under the control of either a CBA promoter (for rAAV1 constructs encoding 14D5-dimHLE, 13A7-IgG1^LSF^ and s+16a-dimHLE) or under the related CASI promoter (for rAAV8 constructs encoding 7E2-IgG2a and s-14-IgG2a) (31, 32). Production, purification, and titration of rAAV1 and rAAV8 were performed by Virovek (Hayward, California, USA) using the baculovirus expression system in Sf9 insect cells (31). For muscle transduction, mice hind legs were shaved under anesthesia and 100 µL diluted rAAV were injected into 4 gastrocnemius muscle sites to reach a total dose of 10^11^ viral genomes (vg) per mouse.

### NAD^+^ Treatment *In Vivo*

Injections of rAAV encoding either 13A7-IgG1^LSF^ hcAb or 14D5-dimHLE were performed i.m. on day 0 and followed 28 days later by the i.v. injection of 30 mg NAD^+^ diluted in 200 μL PBS (pH adjusted to 7.4) as described earlier (24). Splenocytes were collected 24 h later and single-cell suspensions were prepared on ice and stained with fluorochrome-coupled antibodies. After fixation, RBC lysis, permeabilization and intracellular staining, single cell suspensions were analyzed by flow cytometry. Absolute cell numbers collected for each spleen was evaluated by direct enumeration using an automated cell counter (ADAM-MC apparatus). Percentages of CD4^+^ and CD8^+^ T cells were determined by flow cytometry and compared to CD19^+^ B cells taken as an NAD^+^-insensitive reference population.

### Statistical Analysis

All data are shown as mean values and error bars represent standard error of the mean (SEM). Statistical comparison between experimental groups was performed using one-way analysis of variance (ANOVA). Differences were considered statistically significant when p values were less than 0.05 (*), 0.01 (**), or 0.001 (***). All calculations were performed using the Prism software (Graphpad, La Jolla, CA).

## Results

### Nanobody Constructs and rAAV Used to Study the Role of ARTC2.2 and P2X7 *In Vivo*

**Figure 1:**
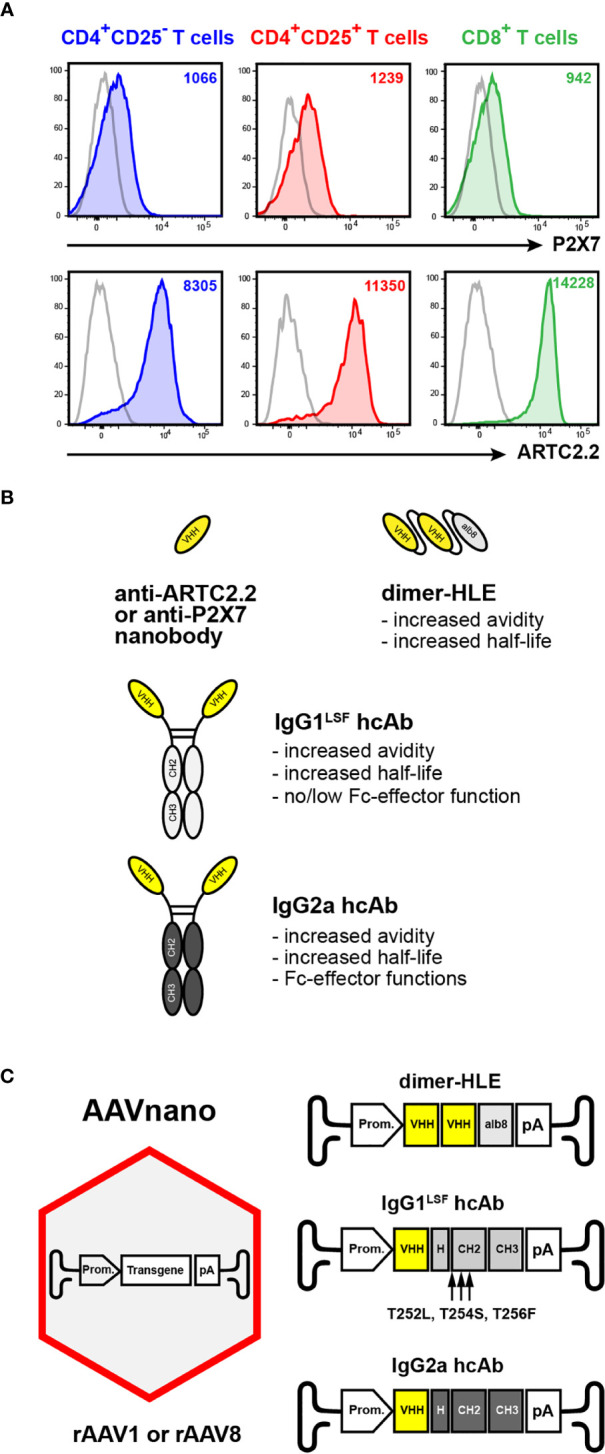
AAVnano methodological approach to study the role of P2X7 or ARTC2.2 *in vivo*. **(A)** Expression of ARTC2.2 and P2X7 T cell subsets. Splenocytes from C57BL/6 mice were stained with fluorochrome-conjugated monoclonal antibodies directed against P2X7 (clone 1F11), or ARTC2.2 (clone Nika102), or with the corresponding isotype controls (grey), and were analyzed by flow cytometry. Cells were gated on CD4^+^CD25^-^ T cells (Tconv, depicted in blue), on CD4^+^CD25^+^ (Treg, depicted in red), or on CD8^+^ T cells (depicted in green) to evaluate the cell surface levels of P2X7 or ARTC2.2 on each subset. The numbers in the upper right quadrants indicate the mean fluorescence intensity (MFI). Staining was performed using fluorochrome conjugated antibodies specific to CD45 (coupled to BV510), CD4 (BV786), CD8 (BV605), CD25 (PE-Cy7), P2X7 (BV421) and ARTC2.2 (AF647). **(B)** Schematic representation of nanobody-based constructs. ARTC2.2-specific and P2X7-specific nanobodies (V_HH_) are engineered either as bivalent dimers fused to the albumin-specific nanobody Alb8 (dimer half-life extended, termed “dimHLE”), or as heavy-chain antibodies (termed hcAb) when fused to the hinge and Fc-region of conventional mouse IgG antibodies. For the latter, nanobodies were fused to either a mouse IgG1 hinge/Fc-region bearing the LSF mutations (*i.e.*, T252L, T254S, T256F) to produce hcAb with no/low Fc-effector functions, or to the hinge/Fc-region of mouse IgG2a to generate cell-depleting hcAb. **(C)** Schematic representation of the AAVnano methodological approach and the structure of the transgenes incorporated in the rAAV (of serotype 1 or 8). The illustrated transgenes encode the nanobody-based constructs depicted in **(B)** Upon a single i.m. injection of the rAAV, the nanobody-based biologics are produced *in vivo* by the transduced muscle cells under the control of a CBA (rAAV1) or a CASI (rAAV8) promoter (Prom.).

The ARTC2.2/P2X7 axis regulates murine T cell function, differentiation and cell fate. Both proteins are expressed on T cell subsets but at different cell surface levels. Consistent with previous studies (12, 13, 24), P2X7 and ARTC2.2 were detected on CD4^+^CD25^+^ Treg, CD4^+^CD25^-^ conventional CD4^+^ T cells (Tconv), and on CD8^+^ T cells. P2X7 is expressed at higher level on the surface of Treg and to a lesser extent on CD4^+^ Tconv and on CD8^+^ T cells (**Figure 1A**, upper panels). ARTC2.2 is expressed at higher levels on CD8^+^ T cells as compared to CD4^+^ T cell subsets (**Figure 1A**, lower panels). To explore the role of P2X7 and ARTC2.2 *in vivo*, we developed a methodological approach based on rAAV encoding various formats of P2X7-specific or ARTC2.2-specific nanobody-based constructs. To increase their half-life and avidity, we developed bivalent nanobodies fused to the albumin-specific nanobody Alb8 (dimer Half-Life Extended format, termed “dimHLE”) (**Figure 1B**). In a second approach, we fused nanobodies to the hinge and Fc region of conventional IgG antibodies (heavy-chain antibodies format, “hcAb”) (**Figure 1B**). Mouse IgG1 Fc subclass is known to only mediate low, if any, effector functions as it binds to the inhibitory FcγRIIB and to only one member of the activating receptors, the low affinity FcγRIII (33). IgG1 also binds to the neonatal FcRn involved in antibody recycling and persistence *in vivo*. To further increase the half-life of our constructs, we took advantage of the described “LSF” point mutations (*i.e.*, T252L, T254S, T256F), known to further ameliorate *in vivo* persistence by increasing the binding to FcRn (30), to generate the mIgG1^LSF^ hcAb construct (**Figure 1B**). In contrast to mouse IgG1, mIgG2a subclass binds all mouse activating FcγR and triggers Fc-related effector functions (**Figure 1B**) (33).

In our methodological approach to target ARTC2.2 and P2X7 on T cells *in vivo*, implemented and evaluated here, we injected rAAV1 or rAAV8 encoding nanobody-based constructs instead of the corresponding purified recombinant proteins. Both rAAV1 and rAAV8 serotypes are known to efficiently transduced muscle cells upon i.m. injection. We aimed at eliciting directly *in situ* the long-term and stable production of the selected construct following a single administration of the corresponding rAAV. This approach was termed AAVnano (**Figure 1C**).

**Figure 2:**
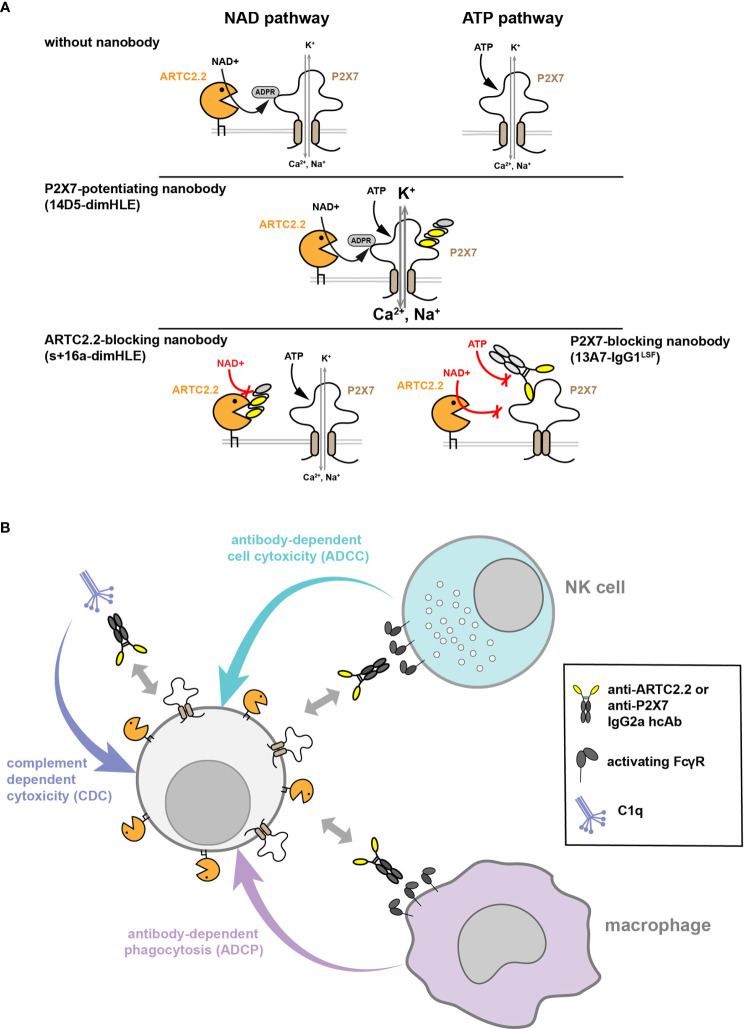
Strategies used to manipulate ARTC2.2 or P2X7 functions *in vivo* or to deplete T cells expressing these proteins. **(A)** Modulation of ARTC2.2 and P2X7 activity using nanobody-based constructs. Two pathways can lead to the activation of P2X7 at the surface of mouse T cells. The ATP pathway involves direct gating of P2X7 ion channel by extracellular ATP. The NAD^+^ pathway involves a post-translational modification of P2X7 catalyzed by the ectoenzyme ARTC2.2 resulting in its covalent ADP-ribosylation. The 14D5-dimHLE construct probably binds to an allosteric site on P2X7 and potentiates gating of the ion channel triggered by either ATP or NAD^+^. The s+16a-dimHLE construct was designed to block ARTC2.2 enzymatic activity *in vivo* and thereby to inhibit activation of P2X7 by the NAD^+^ pathway (but not the ATP pathway). The 13A7-IgG1^LSF^ hcAb construct inhibits P2X7 gating and can be used to inhibit both ATP and NAD^+^ pathways. **(B)** Other nanobody-based constructs were designed to favor depletion of their target cells *in vivo*. For that, nanobodies s-14 and 7E2, that bind respectively to ARTC2.2 and P2X7 but do not modulate their functions, were fused to the hinge and Fc-region of mIgG2a. The resulting mIgG2a hcAb could mediate cell depletion through different mechanisms such as complement-dependent cytotoxicity (CDC), antibody-dependent cell cytotoxicity (ADCC) that rely on NK cells, and antibody-dependent cell phagocytosis (ADCP) involving macrophages.

We explored here two complementary strategies for manipulating *in vivo* the NAD^+^ pathway dependent on ARTC2.2 and P2X7. (**Figure 2**). The first strategy relies on the propensity of nanobodies to bind to functional sites and thereby modulate their enzymatic activity or receptor functions. We used constructs based on nanobodies selected for their remarkable ability to block ARTC2.2 enzymatic activity (nanobody s+16a, used here as a dimHLE format), to inhibit gating of the P2X7 ion channel (nanobody 13A7, used as IgG1^LSF^ hcAb format), or to potentiate P2X7 activity (nanobody 14D5, used as dimHLE format) (**Figure 2A**). The choice of the format for each biological construct (*i.e.*, dimHLE, or IgG1^LSF^ hcAb formats) was based on preliminary experiments comparing the efficacy of each formats (data not shown). The second strategy relies on the possibility to deplete target cells expressing high levels of ARTC2.2 or P2X7. For that, we used hcAb engineered to contain the mIg2a Fc-region, known to promote binding to FcR on immune cells and to promote ADCC by NK cells, ADCP by macrophages, and to facilitate CDC upon engagement on the classical complement pathway (**Figure 2B**). To facilitate the interpretation of their effect *in vivo*, these latter constructs were based on other nanobodies that do not modulate the functional activity of their target (*i.e.*, ARTC2.2-specifc nanobody s-14 (23) and P2X7-specifc nanobody 7E2 (25)) that were fused to the hinge and Fc-region of mouse IgG2a (**Figures 1B, C****)**.

### Functional Modulation of ARTC2.2 or P2X7 Using rAAV Coding for Biologics With Blocking or Potentiating Properties

**Figure 3:**
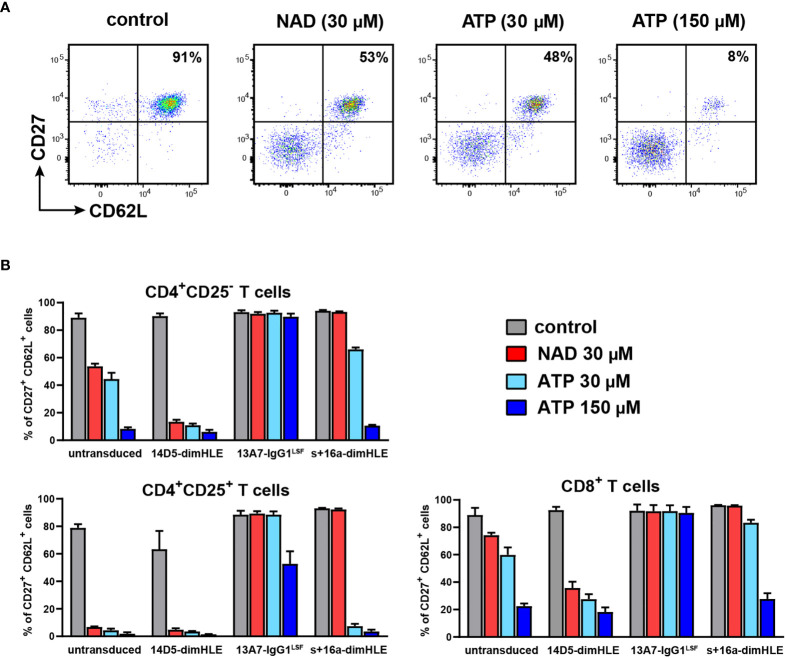
AAVnano modulate P2X7-dependent shedding of CD27 and CD62L *ex vivo*. C57BL/6 mice were injected i.m. with PBS (control) or with rAAV1 encoding P2X7-potentiating 14D5-dimHLE, P2X7-blocking 13A7-IgG1^LSF^ hcAb or ARTC2.2-blocking s+16a-dimHLE. Blood samples were collected at different time points ranging from day 17 to day 120 after rAAV injections. Cells were then incubated for 15 min at 37°C with PBS, 30 µM ATP, 150 µM ATP or 30 µM NAD^+^ to induce P2X7-dependent shedding of CD27 and CD62L on T cells. Representative results obtained 30 days after rAAV injection are shown. **(A)** Representative flow cytometry plots illustrating shedding of CD27 and CD62L on gated CD4^+^CD25^-^ T cells harvested from control mice. Numbers indicate percentages of CD27^+^CD62L^+^ cells. **(B)** Mean percentages of CD27^+^CD62L^+^ cells among gated CD4^+^CD25^+^, CD4^+^CD25^-^ and CD8^+^ T cells after incubation with PBS (grey), 30 µM NAD^+^ (red), 30 µM ATP (cyan) or 150 µM ATP (blue). Staining was performed using fluorochrome conjugated antibodies specific to CD45 (coupled to APC-Cy7), CD4 (FITC), CD8 (PE-Cy7), CD25 (PE), CD27 (PerCP-Cy5.5) and CD62L (APC). Errors bars represent the SEM, n=3.

To evaluate our AAVnano approach and the ability of our constructs to modulate their target proteins, we first indirectly studied the activity of P2X7 and ARTC2.2 on T cells harvested from AAVnano injected mice. Exposure of T cells to ATP or NAD^+^ induces the shedding of CD27 and CD62L by metalloproteases and this can be used as a sensitive assay to monitor P2X7 receptor activation (13, 24). For that, we injected rAAV1 encoding 13A7-IgG1^LSF^ hcAb (*i.e.*, to inhibit P2X7), 14D5-dimHLE (*i.e.*, to potentiate P2X7), or s+16a-dimHLE (*i.e.*, to inhibit ARTC2.2). Blood cells were collected 17-120 days after rAAV injection, incubated at 37°C in the absence or in the presence of NAD^+^ (30 µM) or ATP (30 μM or 150 μM) to assay *ex vivo* P2X7-dependent shedding of CD27 and CD62L. Representative data obtained 30 days after AAVnano injection are shown in **Figure 3**. Comparable results were obtained from the earliest studied time point at day 17 to the last time point of the study at day 120. The results show that T cells from untransduced mice displayed P2X7-dependent shedding of CD27 and CD62L within 15 min after incubation with ATP or NAD^+^ (**Figures 3A, B****)**. This was noticeably more pronounced on CD4^+^CD25^+^ Treg that express higher levels of P2X7 as compared to Tconv and to CD8^+^ T cells (**Figure 3B**). Injection of AAVnano coding for 14D5-dimHLE sensitized T cells to P2X7-dependent shedding, notably also on the T cell subsets that express the lowest levels of P2X7 (*i.e.*, Tconv and CD8^+^ T cells) as compared to untransduced control mice. In contrast, mice injected with the AAVnano coding for 13A7-IgG1^LSF^ hcAb were almost completely protected from NAD^+^- and ATP-induced P2X7 activation, and only Treg cells displayed limited CD27 and CD62L shedding when incubated with the highest dose of ATP (**Figure 3B**). T cells from mice injected with the AAVnano coding for s+16a-dimHLE were protected from NAD^+^-induced, but not from ATP-induced shedding of CD27 and CD62L, as expected from a nanobody construct that inhibits ARTC2.2 activity (**Figure 3B**). For comparison, we also evaluated the effects mediated by recombinant nanobody-based biologics added directly *in vitro* on cells from untreated animals (**Supplemental Figure 1**). We observed in dose-response experiments that each recombinant construct used at saturating doses *in vitro* induced a comparable effect that the one observed *ex vivo* on cells collected from AAVnano transduced mice. These data hence suggest that i.m. injection of AAVnano induce *in vivo* production of each encoded construct in sufficient quantity to substantially inhibit or potentiate the functional activity of their target, at a level that appears to be similar to the one obtained *in vitro* with saturating doses of each given recombinant nanobody construct.

### Evaluation of Tissue Resident T Cells *In Vivo* Following Administration of rAAV Coding for Biologics

**Figure 4:**
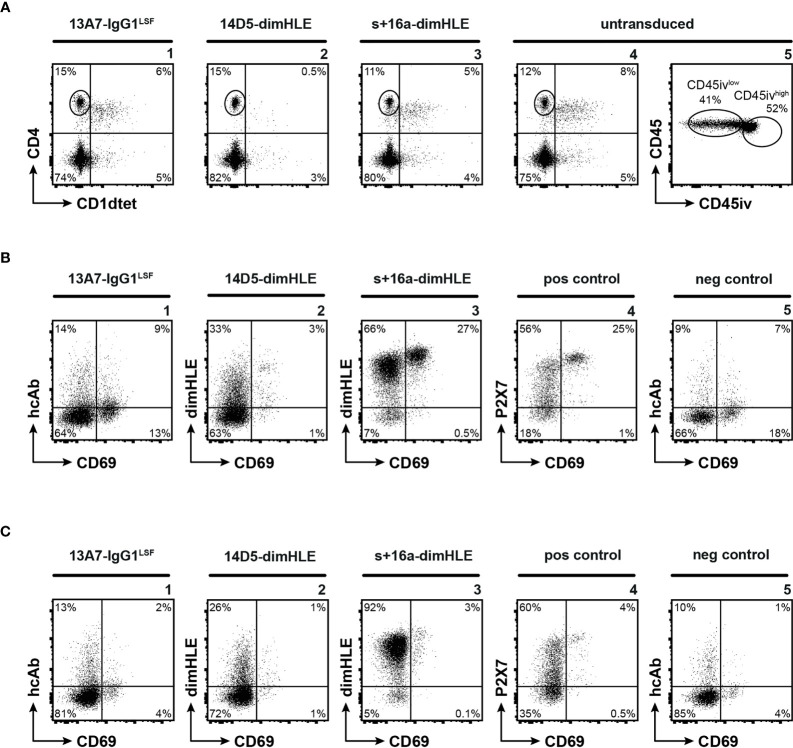
Nanobody-based biologics produced *in vivo* following rAAV injection reach ARTC2.2 and P2X7 expressed at high levels by T_RM_ located in the liver parenchyma. C57BL/6 mice were injected i.m. with rAAV1 encoding P2X7-blocking 13A7-IgG1^LSF^ hcAb, P2X7-potentiating 14D5-dimHLE, or ARTC2.2-blocking s+16a-dimHLE, or with PBS (untransduced). 120 days after rAAV injection mice received an intravenous injection of fluorochrome-conjugated CD45-specific mAb (CD45iv) 3 min before sacrifice. Untransduced mice additionally received 50 µg of recombinant s+16a-dim construct 30 min before organ collection to prevent NICD and cell loss *ex vivo* during cell preparation, as described earlier (15, 18). Cells extracted from the liver were then counterstained with mAbs directed against CD45, CD4, and CD69 (i.e., a marker of tissue resident lymphocytes) and with a CD1d-tetramer (CD1dtet) loaded with αGal/Cer (specifically labels the invariant T cell receptor of NKT cells). **(A)** Representative flow cytometry plots illustrating liver CD4^+^ T cells and CD4^+^CD1dtet^+^ NKT cells (panels 1-4), and the gating of parenchymal (CD45iv^low^) and vascular (CD45iv^high^) CD4^+^CD1dtet^-^ lymphocytes (panel 5). **(B, C)** Representative flow cytometry plots illustrating the detection of the nanobody-based constructs on the cell surface of tissue resident CD45iv^low^ **(B)** and vascular CD45iv^high^ **(C)** CD4^+^CD1dtet^-^ T cells. To detect cell surface bound dimHLE constructs, cells were stained with Alb8-specific mAb77 (mouse IgG1) followed by an mIgG1-specific secondary mAb. To detect cell surface bound 13A7-IgG1^LSF^, cells were stained directly with the secondary mIgG1-specific mAb. Positive (pos) control staining was performed with liver cells from untransduced mice using the P2X7-specific 13A7-dimHLE, mAb77, and the mIgG1-specific secondary mAb. Negative (neg) control staining was performed with the secondary detection reagents alone. Numbers indicate the percentages of cells in the respective quadrants or gates (**A**, panel 5). Staining was performed using fluorochrome conjugated antibodies specific to CD45 (coupled to PerCP-Cy5.5 for the one injected i.v. and to AF700 for the one added *in vitro*), CD4 (APC), CD69 (FITC), mouse IgG1 (BV421) and CD1dtet (coupled to PE).

To determine whether our methodological approach could be used to reach ARTC2.2 and P2X7 on tissue resident T cells, we next sought to detect the presence of the biologics on resident liver T cells 120 days after rAAV injection. For that, we focused at the end of the experiment (*i.e.*, upon sacrifice of animals) on liver tissue-resident memory T cells (T_RM_) and for comparison on liver vascular T cells as well. Liver T_RM_ co-express higher levels of ARTC2.2 and P2X7 than conventional T cells (18) (**Figure 4**). In order to discriminate tissue resident from vascular lymphocytes, we intravenously injected a fluorochrome-conjugated CD45-specific mAb 3 min before sacrifice. In this short period of time, the injected antibody stains vascular, but not tissue resident lymphocytes (34). In order to detect the rAAV-encoded s+16a-dimHLE or 14D5-dimHLE, we used a monoclonal antibody (mAb77) that specifically recognizes the Alb8 nanobody, and for detection of 13A7-IgG1^LSF^, we used a mouse IgG1-specific secondary antibody. We further stained cells with a CD4-specific mAb and an αGal/Cer-loaded CD1d tetramer to distinguish NKT cells from other CD4^+^ T cells. NKT cells are also known to co-express high levels of ARTC2.2 and P2X7 and to be highly sensitive to NAD^+^ induced cell death (NICD) (18). Cells were counterstained with a fluorochrome-conjugated mAb against CD69, a marker that is highly expressed by many tissue resident T cells but by only few if any vascular T cells (35, 36). Untransduced mice were used as control and were given recombinant s+16a-dim 30 min before sacrifice to prevent NICD and cell loss during preparation as described before (15, 18). The results confirmed the higher level of ARTC2.2-specific s+16a-dimHLE bound at the surface of CD69^+^ T_RM_ as compared to tissue resident CD69^-^ CD4^+^ T cells and to vascular CD4^+^ T cells (**Figures 4B, C**, panels 3). Also, higher expression of P2X7 was detected at the surface of CD69^+^ T_RM_ compared to other CD4^+^ subsets (**Figures 4B, C** panels 4). In mice injected with rAAV1 encoding the ARTC2.2-specific s+16a-dimHLE, we observed high proportions of NKT cells and CD69^+^ T_RM_ as expected from an ARTC2.2-blocking biologics that protect from NICD that occurs during cells preparation (**Figures 4A, B**, panels 3). In contrast, liver lymphocytes obtained from mice injected with rAAV1 encoding the P2X7-potentiating 14D5-dimHLE show markedly reduced percentages of tetramer-stained NKT cells and of CD69^+^ T_RM_ (**Figures 4A, B**, panels 2), *i.e.* a cell populations that display high cell surface level of P2X7 (**Figure 4B**, panel 4). This likely reflects 14D5-dimHLE enhanced cell death of these subsets due to NAD^+^ released during liver collection and cell preparation (34). Liver lymphocytes recovered from mice injected 120 days earlier with rAAV1 encoding the P2X7-blocking 13A7-IgG1^LSF^, contained high proportion of NKT cells (**Figure 4A**, panel 1) and of CD69^+^ T_RM_ (**Figure 4B**, panel 1), indicating that binding of 13A7-IgG1^LSF^ rendered these subpopulations resistant to NICD during cell preparation. However, the low level of staining with the IgG1-specific secondary antibody (**Figure 4B**, panel 1) suggests that this hcAb induced down modulation of cell surface P2X7, possibly by hcAb-induced endocytosis. This was not due to low binding of 13A7-IgG1^LSF^ at the surface of these cells as incubation of the cells *ex vivo* with a saturating dose of recombinant 13A7-IgG1^LSF^ followed by IgG1-specific secondary antibody did not increased the staining (data not shown). Similarly, in mice injected with rAAV1 encoding 14D5-dimHLE or s+16a-dimHLE, incubation of cells *ex vivo* with a saturating dose of the corresponding recombinant protein, followed by the appropriate secondary detection reagent, did not increased the staining intensity (not shown). This data indicate that each of these nanobody-based constructs produced *in vivo* achieved full occupancy of their respective target proteins expressed on vascular and tissue resident CD4^+^ T cells, as expected from their continuous endogenous production mediated by rAAV1 injection 120 days earlier.

### Functional *In Vivo* Modulation of ARTC2.2 or P2X7 by rAAV Encoded Biologics

**Figure 5:**
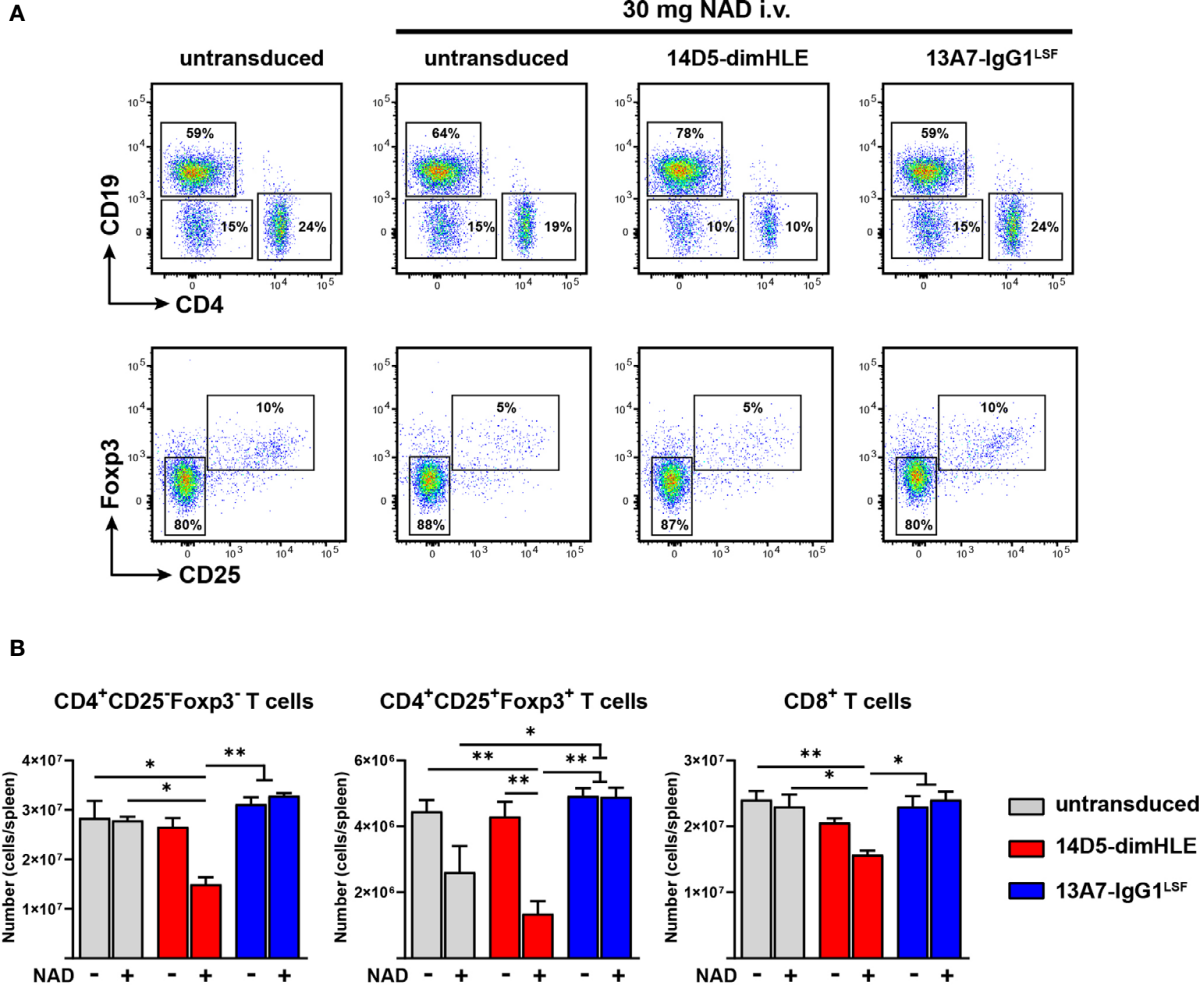
AAVnano modulate *in vivo* the sensitivity to NAD^+^-induced cell death. C57BL/6 mice were injected i.m. with PBS (control) or with rAAV1 encoding the P2X7-potentiating 14D5-dimHLE construct or the P2X7-blocking 13A7-IgG1^LSF^ hcAb construct. 28 days later mice were injected i.v. with PBS or 30 mg NAD^+^. Splenocytes were collected one day after and analyzed by flow cytometry to evaluate the levels of P2X7-dependent T cell depletion induced by NAD^+^ injection. **(A)** Representative flow cytometry plots showing the relative percentages of cells in each group. In the upper panels, CD4^+^, CD8^+^ and CD19^+^ lymphocytes were concatenated in the same subpopulation and analyzed together to visualize the level of depletion of CD4^+^ and CD8^+^ (CD4^-^/CD19^-^) T cells as compared to NAD^+^-insensitive CD19^+^ B cells. In the lower panels, the gated CD4^+^ cells were analyzed for both, expression of Foxp3 and CD25 to evaluate the percentages of Treg depletion in each group. Numbers indicate the percentage of cells in each indicated gate. **(B)** Absolute numbers of cells collected from spleen of untransduced control mice (grey) or from mice injected with rAAV encoding 14D5-dimHLE (red) or for 13A7-IgG1^LSF^ hcAb (blue). Each group was injected i.v. either with PBS (-) or with 30 mg of NAD^+^ (+). Error bars represent the SEM. The statistical analysis was performed using one-way ANOVA (n=3 in each group, *p < 0.05, **p < 0.01). Staining was performed using fluorochrome conjugated antibodies specific to CD45 (coupled to BV510), CD4 (APC-Cy7), CD8 (BV605), CD19 (PerCP-Cy5.5), CD25 (PE) and FoxP3 (BV421).

We next evaluated the effect of these biological constructs *in vivo*. First, we confirmed the absence of cell depletion using these AAVnano by evaluating the percentages of CD4^+^CD25^+^ Treg, CD4^+^CD25^-^ Tconv, and CD8^+^ T cells, throughout the study (data not shown). We then injected NAD^+^ i.v. and evaluated *in vivo* the modulating effect of the P2X7-specific constructs. As we previously demonstrated that the injection of 60 mg NAD^+^ provokes the depletion of 80% of Treg (24), we injected here a limited dose of 30 mg of NAD^+^ in order to evaluate the potentiating effect of 14D5-dimHLE. For that, mice were injected again with each AAVnano, followed 28 days after by the i.v. injection of 30 mg NAD^+^. Depletion of cell subsets was evaluated by collection and analyses of splenocytes one day after. As expected, untransduced mice showed a limited yet significant depletion of splenic Treg (**Figures 5A, B****)**, but not of other T cell subsets that express lower levels of P2X7. In contrast, mice injected with the AAVnano encoding 13A7-IgG1^LSF^ hcAb were protected from NAD^+^-induced Treg depletion. Remarkably, mice injected with the vector encoding the P2X7-potentiating construct 14D5-dimHLE showed significantly enhanced cell depletion, not only for Treg but also for the less sensitive Tconv and CD8^+^ T cell subsets, confirming the potentiating effect observed *ex vivo* (**Figures 3B**, **5B**). These data provide evidence that P2X7 activity at the surface of T cell subsets can be modulated *in vivo* following a single injection of a rAAV1 encoding blocking or potentiating anti-P2X7 nanobody-based biologics.

### Depletion *In Vivo* of Cells Expressing ARTC2.2 or P2X7 Using rAAV Coding for Nanobody-Based hcAb

**Figure 6:**
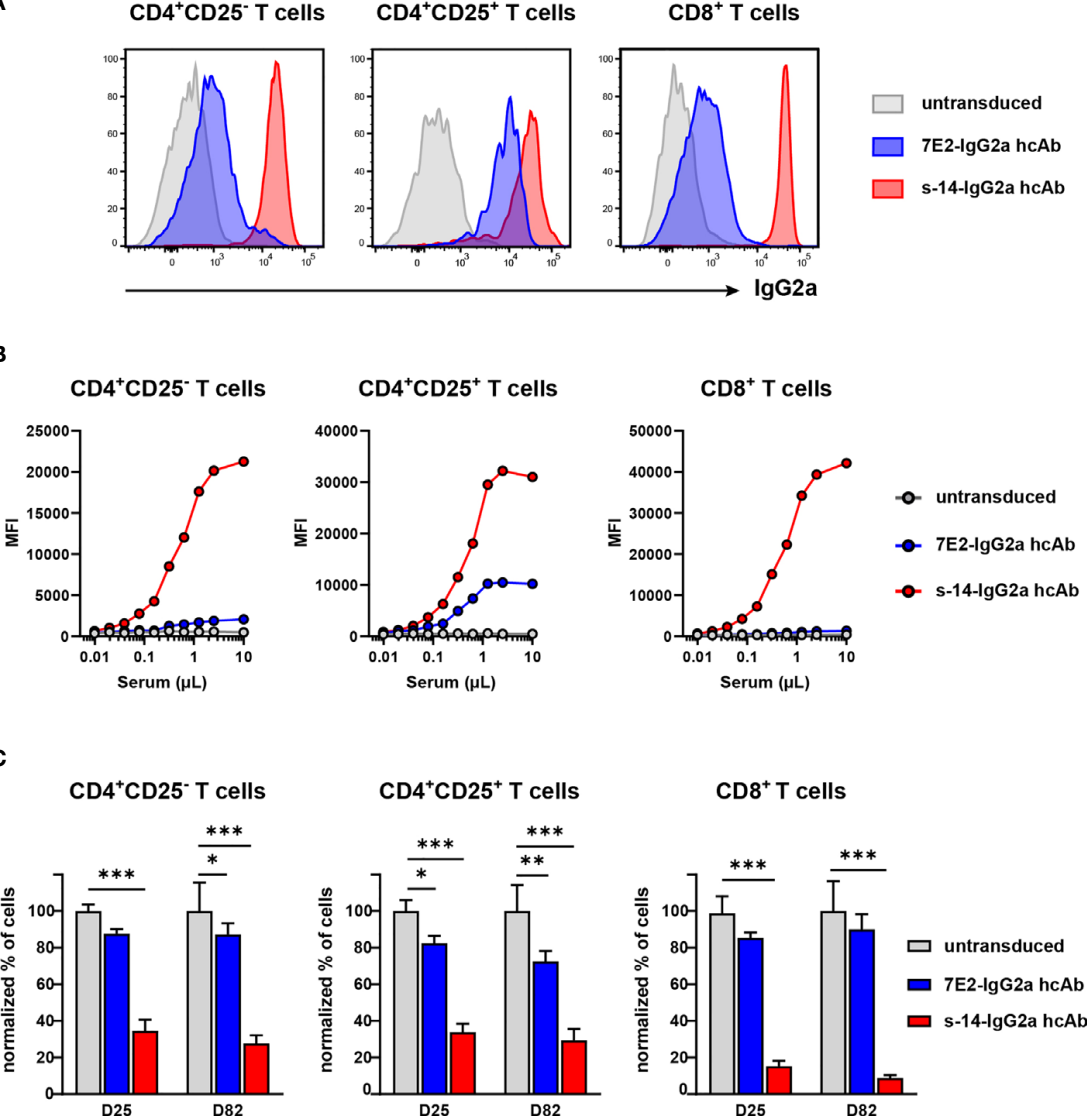
AAVnano encoding nanobody-based IgG2a hcAb mediate depletion of cells expressing high levels of P2X7 or ARTC2.2. C57BL/6 mice were injected with PBS (untransduced, grey) or with rAAV8 encoding 7E2-IgG2a (blue) or s-14-IgG2a (red) hcAb. **(A, B)** Sera were collected 63 days post rAAV8 i.m. injection. To monitor the levels of hcAb in serum, splenocytes from an untreated naive C57BL/6 mouse were incubated with diluted serum, washed and bound hcAb were detected with an IgG2a-specific secondary antibody. **(A)** Flow cytometry analyses illustrating the binding of each nanobody-based IgG2a hcAb on the indicated subset of splenic cells from an untreated mouse when using 10 μl of pooled sera collected from rAAV injected mice. **(B)** Splenocytes from an untreated mouse were incubated with serial dilutions of sera to titrate the relative abundance of the two hcAb. For both hcAb, serum volumes between 1-10 µL were enough to saturate the signal. **(C)** To evaluate depletion of T cell subsets *in vivo*, blood samples were collected 25 days and 82 days after i.m. injection of rAAV encoding the indicated IgG2a hcAb. Cells were stained with fluorochrome-conjugated antibodies and analyzed by flow cytometry. Normalized percentage of CD4^+^CD25^-^, CD4^+^CD25^+^ and CD8^+^ T cells are shown, as compared to the percentages of cells found in the control group taken as 100%. Staining was performed using fluorochrome conjugated antibodies specific to CD45 (coupled to PerCP-Cy5.5), CD4 (APC-Cy7), CD8 (PE-Cy7), CD25 (PE) and biotinylated antibody to mouse IgGa and streptavidin-APC. Errors bars represent the SEM and statistical analyses were performed using one-way ANOVA (n=7 in each group, *p < 0.05, **p < 0.01, ***p < 0.001).

Upon binding, conventional antibodies can promote target cell depletion through their Fc-related effector functions. We aimed here at evaluating P2X7-specifc and ARTC2.2-specific hcAb that contain a neutral nanobody (*i.e.*, a nanobody that bind to its target but does not modulate its function) fused to the mouse IgG2a Fc-region, for their capacity to promote cell depletion *in vivo*. For that, we used again rAAV coding for these constructs that we termed 7E2-IgG2a hcAb for the one targeting P2X7, and s-14-IgG2a hcAb for the one directed against ARTC2.2. First, we evaluated the binding of each of these constructs to the surface of mouse T cell subsets. For that, serum from mice injected 63 days earlier with hcAb-encoding rAAV8 were collected and used as a source of hcAb. These sera were then incubated with splenocytes harvested from untreated naive mice and bound hcAb were detected by a IgG2a-specific secondary antibody (**Figure 6A**). Results confirmed higher binding of s-14-IgG2a hcAb on the surface of the CD8^+^ T cell subsets in agreement with the higher expression of its ARTC2.2 target on this subset (**Figure 1A**). 7E2-IgG2a hcAb was found to bind at lower levels than s-14-IgG2a hcAb at the surface of each studied T cell subsets, in agreement with the lower expression of P2X7 as compared to ARTC2.2 on these cell populations. Yet, 7E2-IgG2a hcAb was found to bind better on Treg than on Tconv and CD8^+^ T cells (**Figure 6A**), in agreement with the higher expression of P2X7 on Treg (**Figure 1A**). Titration analyses indicated that around 1 μl of serum contained saturating amounts of hcAb (**Figure 6B**). These results indicate that 7E2-IgG2a and s-14-IgG2a hcAb are produced in saturating amounts *in vivo* at least 63 days following a single i.m. injection of rAAV.

For comparison, and to evaluate the kinetic and relative abundance of hcAb production *in vivo*, we also used sera (or plasma) collected at different time points from another experiment to stain HEK293-ARTC2.2 or HEK293-P2X7 cell lines expressing high and stable levels of surface ARTC2.2 or P2X7, obtained by retroviral transduction of the parental HEK-293 cell line (**Supplemental Figure 2**). The data confirm that 7E2-IgG2a hcAb and s-14-IgG2a hcAb are detectable in the circulation at the earlier time point studied (i.e., at day 13 post rAAV8 i.m. injection), are produced in all animals that were studied at a rather similar level, and slowly accumulate to reach a maximum concentration between 45 and 66 days post rAAV8 injection and remains stable thereafter (**Supplemental Figure 2**).

We next analyzed the level of target cell depletion overtime induced by the production of ARTC2.2-specific or P2X7-specific hcAb *in vivo*. Results obtained with the P2X7-specific 7E2-IgG2a hcAb showed a modest depletion of Treg, reaching 18 ± 4% at day 25 post rAAV injection. The proportions of CD4^+^CD25^-^ Tconv and CD8^+^ T cells were only slightly decreased in mice producing 7E2-IgG2a hcAb, consistent with lower levels of P2X7 on these subsets (**Figures 6A–C**). In contrast, in mice producing ARTC2.2-specific s-14-IgG2a hcAb, we found a substantial depletion of the three T cell subsets analyzed. Depletion was most pronounced for CD8^+^ that express the highest level of ARTC2.2. This construct was indeed found to deplete 85 ± 3% of CD8^+^ T cells, 65 ± 6% of Tconv, and 66 ± 5% of Treg 25 days post rAAV8 injection. The level of depletion was slightly higher at day 82 post rAAV8 injection (**Figure 6C**). For comparison, CD19^+^ B cells (that do not express P2X7 nor ARTC2.2) and myeloid non-granulocytic CD11b^+^ cells (that do express very little, if any, ARTC2.2 and low level of P2X7) were also enumerated in the blood at the same time point. Data demonstrated the absence of depletion of these subsets in agreement with their absence of expression or low expression of ARTC2.2 and/or P2X7 (**Supplemental Figure 3**). These findings illustrate the long-term and stable depleting effects of the target T cells elicited by these IgG2a hcAb upon a single i.m. injection of the corresponding hcAb-encoding rAAV8.

## Discussion

Surface expression of the ectoenzyme ARTC2.2 is restricted to T cells and is found at higher levels on murine CD8^+^ T cells as compared to CD4^+^CD25^+^ Treg or CD4^+^CD25^-^ Tconv (**Figure 1A**) (9). P2X7 is expressed by multiple cells, including immune cells of the myeloid and lymphoid compartments. On murine T cells, P2X7 is differentially expressed on T cell subsets, and was found to be expressed at higher levels on NKT cells, Treg, Tfh, and T_RM_ (12, 14–18). The homotrimeric P2X7 receptor is known to form an ATP-gated ion-channel connected to multiple signaling pathways that regulate cell phenotype, functions, differentiation and survival (11). For murine T cells, P2X7 activation can be triggered by two complementary pathways. As in myeloid and non-immune cells, the P2X7 receptor can be gated by the presence of relatively high concentrations of extracellular ATP (*i.e.*, 30 to 300 µM range) that can be released from dying or stressed cells. In addition, in murine T cells, extracellular NAD^+^ was found to trigger activation of the P2X7 receptor at low micromolar concentrations (12, 24). The mechanism involves the ARTC2.2 catalyzed covalent ADP-ribosylation of P2X7 on R125 in the vicinity of the ATP-binding site (23). Although much was learnt during the last years on the multifaceted P2X7 receptor, the relative contribution of the ATP and NAD^+^ pathways their activation *in vivo*, their precise immunoregulatory roles, and their contribution in pathophysiological conditions, remain to be better addressed and studied. The AAVnano approach described here uses rAAV for durable production of nanobody-containing biologics *in vivo*. This could be used in future studies to better delineate ARTC2.2 and/or P2X7 functions in animal models of acute as well as chronic diseases.

Our results show that a single injection of rAAV1 or rAAV8 encoding P2X7-specific or ARTC2.2-specific nanobody-based constructs (**Figure 1**) can inhibit or in contrast potentiate ATP- or NAD^+^-induced activation of P2X7 *ex vivo* on splenic T cells harvested from these mice. As one hallmark associated with P2X7 receptor activation, we used a sensitive *ex vivo* assay based on metalloprotease-dependent shedding of CD27 and CD62L from the T cell surface. Our data demonstrate that a single injection of a rAAV encoding the P2X7-blocking 13A7-IgG1^LSF^ hcAb protects T cells from NAD^+^- as well as ATP-induced CD27 and CD62L shedding (**Figure 3B**). Similarly, injection of a rAAV encoding the ARTC2.2-blocking s+16a-dimHLE completely inhibits NAD^+^- but not ATP-induced shedding of CD27 and CD62L (**Figure 3B**). Remarkably, the P2X7-potentiating 14D5-dimHLE sensitized T cells to NAD^+^- as well as ATP-induced shedding of CD27 and CD62L. This is noticeable also for Tconv and CD8^+^ T cells that express low levels of P2X7 and are therefore less sensitive to activation of P2X7 than Treg (**Figure 3B**).

Analysis of vascular and tissue resident liver T cells from mice sacrificed 120 days after injection of AAVnano revealed full occupancy of ARTC2.2 by s+16a-dimHLE on CD4^+^ T cells in the vasculature and liver parenchyma (**Figures 4B, C** and data not shown). Moreover, the high recovery of CD69^+^ T_RM_ indicates that this cell population is fully protected from NAD-induced cell death (NICD) during cell preparation in these mice (**Figure 4B**). Much lower numbers of NKT cells and CD69^+^ T_RM_ (*i.e.*, the cell populations with the highest cell surface levels of P2X7) were recovered from mice injected with the rAAV1 encoding 14D5-dimHLE, consistent with its P2X7-potentiating activity that might enhance cell death *in vivo* and/or during cell preparation. High numbers of liver T_RM_ were recovered from mice expressing *in vivo* the P2X7-blocking 13A7-IgG1^LSF^ hcAb, consistent with its protective role on NICD. The low staining of these cells with the IgG1-specific secondary Ab, possibly reflects hcAb induced endocytosis of P2X7 by these cells.

We next evaluated directly *in vivo* the potential of these endogenously produced constructs to modulate P2X7 receptor activation. We previously reported that a single i.v. injection of 60 mg NAD^+^ depletes about 80% of CD4^+^CD25^+^Foxp3^+^ Treg following ARTC2.2-dependent activation of P2X7 (24). We injected here a lower dose of 30 mg NAD^+^ to better evaluate the potentiating effect of 14D5-dimHLE. The results indeed demonstrate that this dose depletes 41 ± 18% of the Treg cells in untransduced control mice, while 68 ± 9% Treg cells are depleted by the same treatment in animals whose muscle cells had been transduced with rAAV1 coding for 14D5-dimHLE (**Figure 5B**). Even the less sensitive Tconv and CD8^+^ T cells, which were not affected by 30 mg NAD^+^ in untransduced mice, were significantly depleted in animals expressing 14D5-dimHLE (although to a lesser degree than Treg). These results are consistent with the data obtained *ex vivo*, that 14D5-dimHLE potentiates P2X7 function also in T cell subsets that display lower P2X7 surface expression. Taken together, these data demonstrate that the AAVnano approach described here is a feasible approach to reproducibly modulate ARTC2.2 or P2X7 functional activities *in vivo*.

Several small molecules P2X7 blockers have been developed by pharmaceutical and biotechnology companies in the past years since P2X7 is a potential target in inflammatory diseases and in cancer (37–41). Several studies have indeed evidenced that pharmacological blockade of P2X7 is associated with therapeutic benefits in pre-clinical animal models of inflammation, pain, autoimmune and neurodegenerative diseases, and cancer. Given that many cancer cells express high levels of P2X7 receptors (notably mutated, truncated, or splice variants that are not able to trigger P2X7-dependent cell death), and that its tonic activation in the tumor microenvironment is associated with tumor proliferation and invasiveness, P2X7-blockade was envisioned as a possible cancer therapy. This was demonstrated in several animal studies (42, 43). Using our AAVnano approach, coding for the P2X7-blocking 13A7-IgG1^LSF^ hcAb, we recently reported a similar finding (44). This suggests that the AAVnano methodological approach, in addition to knock-out models and small molecule inhibitors, represents an alternative and complementary possibility to validate *in vivo* the importance of P2X7 target in various animal models.

In addition to P2X7-blockers, some compounds were recently identified as positive allosteric modulators of P2X7 activation (45–47). As for the 14D5-dimHLE biologics described here, these molecules offer the interesting perspective to potentiate P2X7 only in the microenvironment *in vivo* where NAD^+^ and/or ATP are present in the extracellular space in sufficient quantities to trigger P2X7-gating, as for instance in the tumor microenvironment (48). One such small-molecule positive allosteric modulator of P2X7, named HEI3090, has recently shown promising results in mouse models of non-small cell lung cancer and melanoma (49). Interestingly, the mechanism involves the stimulation of immune cells (*i.e.*, dendritic cells, NK cells, and CD4^+^ effector T cells), and production of IL-18. This finding underscores the notion that P2X7 stimulation may indeed be beneficial in certain circumstances. Since both, blocking or potentiating P2X7 seems to be beneficial in some cancer models, the AAVnano approach described here, allowing both modalities to be studied using the same approach, might be used in future studies as a tool to extend our knowledge on the positive and negative functions of P2X7 in different disease models.

Another potential application that we explored here is the development and use of nanobody-based hcAb to promote cell depletion *in vivo*. We exploited the natural ability of Fc-region to bind to FcR on immune cells and to mediate Fc-related effector functions and/or to induce the activation of the classical complement pathway. For that, we fused P2X7-specific or ARTC2.2-specific nanobodies to the hinge and Fc region of mouse IgG2a to generate nanobody-based hcAb. We then evaluated the capacity of these biologics to mediate the depletion of target-expressing cells *in vivo* following i.m. injection of rAAV8 that encode these constructs. In line with the cell surface levels of ARTC2.2 and P2X7 on T cell subsets, our results revealed that the ARTC2.2-specific s-14-IgG2a hcAb mediated considerably higher cell depletion than the P2X7-specific 7E2-IgG2a hcAb (**Figure 6C**). Also, when considering each construct individually, the relative level of cell depletion of each T cell subset was found to correlate with the relative abundance of the target protein at the cell surface. Thus, the ARTC2.2-specific s-14-IgG2a hcAb depleted the CD8^+^ subset more efficiently than the other two subsets (**Figures 6A, B****)**. Similarly, although at lower levels, the P2X7-specific 7E2-IgG2a hcAb depleted Treg more efficiently than the other two subsets (**Figures 6A–C**). Hence, cell depletion appeared in our models to depend on the level of the target antigen at the surface of each T cell subset. Higher target antigen levels might indeed promote higher FcγR cross-linking on the surface of effector cells, as well as a higher propensity to bind C1q and to activate the complement cascade. However, we cannot exclude that other factors may contribute to the differences observed in the level of depletion with these two hcAb. Factors such as the location of the epitope recognized by the nanobody, the lateral mobility of the target protein at the cell surface (*i.e.*, ARTC2.2 is GPI-anchored protein while P2X7 is a two-transmembrane domain protein), its degree of oligomerization (ARTC2.2 is a monomer while P2X7 forms a homotrimeric ion channel), may also influence the level of cell depletion. Nonetheless, high levels of cell depletion that reached up to 85% of CD8^+^ cells were reproducibly obtained *in vivo* using the rAAV coding the ARTC2.2-specific s-14-IgG2a hcAb and this level of depletion was remarkably maintained throughout the study until the latest time point studied (**Figure 6C**). We propose that this strategy may be used to deplete cells that express the highest levels of ARTC2.2 *in vivo* and provides a tool for studying the NAD^+^/ARTC2.2 signaling pathways. In parallel, increasing the depleting efficiency of P2X7-specific hcAb may also be of interest for translational preclinical cancer studies. As mentioned earlier, P2X7 is expressed at the surface of many cancer cells. Depleting P2X7-specific hcAb may thus be used directly to eliminate tumor cells and additionally to deplete Treg (*i.e.*, T cells expressing higher levels of P2X7) potentially resulting in the induction of two synergistic anti-tumor mechanisms.

In this proof-of-principle study, we illustrated the potential of two methodological approaches to study ARTC2.2 and P2X7 function in animal model using rAAV. The aim of AAVnano is to bypass the need to produce and characterize recombinant proteins *in vitro*, to avoid protein injections every 1-2 days, and to favor a stable concentration and bioavailability. We demonstrate that a single i.m. injection of a rAAV encoding a nanobody-based biologic was sufficient to elicit long-term modulation of ARTC2.2 or P2X7 activity, or depletion of the cells expressing these proteins at high cell density. We propose that the AAVnano methodology may be used in future studies for further evaluating the role of ARTC2.2 and/or P2X7 in animal models of various diseases where these proteins have been implicated.

Although this methodological approach have many advantages, including the possibility to explore in parallel potential long-term side effects *in vivo* (i.e, on-target long-term effects as well as off-target effects), AAVnano have also some limitations. Considering the potential long-term side effects, animals remained healthy in our studies during the entire observation period (until day 120) and we did not observed any conspicuous signs of disease or side effects. We cannot however ruled-out that the biologics that were evaluated here induce other effects that we did not fully explored. The P2X7-specific hcAb that we evaluated here may for instance deplete or influence other cells than the studied T cells as for instance myeloid cells or microglial cells known to also express P2X7. Even if we did not notice any significant depletion of CD11b^+^ myeloid cells in blood during our analyses (**Supplemental Figure 3**), more detailed investigations will be required in future studies to evaluate this possibility, notably in tissue-resident cells as well as in diseased animal models. Apart from unanticipated side effects, one obvious limitation of our AAVnano approach is the difficulty to evaluate the candidate nanobody-based biologics in therapeutic schemes. Indeed, AAV-based transgenic expression *in vivo* usually require 10 to 14 days before the biologics can be significantly produced and detected in the circulation. This period can however be reduced to 2 to 4 days using a “self-complementary” (sc) transgene instead of the “single-stranded” (ss) transgene packaged in classical rAAV vectors. Production and use of scAAV may represent therefore an interesting alternative to the ssAAV used in this study when rapid production of the biologics is required (as in therapeutic protocols or for treating tumors). Hence, AAVnano represents at least a method of choice in chronic situations and as a first *in vivo* approach to evaluate efficacy and absence of long-term side effects before evaluation of the corresponding selected recombinant biologics in therapeutic and translational models.

Interestingly, the rAAV encoding P2X7-specific constructs described here represent to our knowledge the first tools that can be used *in vivo* to either inhibit or potentiate the P2X7 receptor durably, offering the possibility of evaluating the role of P2X7 in various pathophysiological animal models. This methodical approach may be particularly promising for the reevaluation of the role of P2X7 in cancer as well as in inflammatory and neurodegenerative diseases where conflicting results have been obtained so far using knock-out models and/or repetitive administration of small-molecule inhibitors.

## Data Availability Statement

The raw data supporting the conclusions of this article will be made available by the authors, without undue reservation.

## Ethics Statement

The animal study was reviewed and approved by Comité d’Ethique NOrmandie en Matière d’EXpérimentation Animale (CENOMEXA).

## Author Contributions

FK-N and SA, conceptualization. HG, MD, RH, AS, MJ, CP-E, FK-N, and SA, methodology. HG, MD, RH, AS, MJ, CP-E, and SA, investigation. HG, FK-N, and SA, writing – original draft. HG, MD, RH, AS, FK-N, OB, and SA, writing – review and editing. FK-N and SA, funding acquisition. RV, OB, FK-N, and SA, resources. FK-N and SA, supervision. All authors contributed to the article and approved the submitted version.

## Funding

This work was supported by a grant from the Deutsche Forschungsgemeinschaft (DFG) to FK-N (No310/13, No310/14, and SFB1192-B5), by a stipend from the Werner-Otto Foundation to MJ, and by a grant from the Agence Nationale de la Recherche (ANR) to SA (ANR-18-CE92-0046).

## Conflict of Interest

The authors declare that the research was conducted in the absence of any commercial or financial relationships that could be construed as a potential conflict of interest.

## Publisher’s Note

All claims expressed in this article are solely those of the authors and do not necessarily represent those of their affiliated organizations, or those of the publisher, the editors and the reviewers. Any product that may be evaluated in this article, or claim that may be made by its manufacturer, is not guaranteed or endorsed by the publisher.

## Abbreviations

NAD^+^, nicotinamide adenine dinucleotide; ART, ADPribosyltransferase; hcAb, heavy chain antibody; dimHLE, dimer half-life extended; rAAV, recombinant adeno-associated virus used as a vector; AAVnano, methodological approach based on the injection of rAAV encoding for nanobody-based biologics.

## References

[1] Koch-Nolte F Haag F Guse AH Lund F Ziegler M. Emerging Roles of NAD+ and its Metabolites in Cell Signaling. Sci Signal (2009) 2:mr1. 10.1126/scisignal.257mr1 19211509

[2] Linden J Koch-Nolte F Dahl G. Purine Release, Metabolism, and Signaling in the Inflammatory Response. Annu Rev Immunol (2019) 37:325–47. 10.1146/annurev-immunol-051116-052406 30676821

[3] Adriouch S Haag F Boyer O Seman M Koch-Nolte F. Extracellular NAD+: A Danger Signal Hindering Regulatory T Cells. Microbes Infect (2012) 14:1284–92. 10.1016/j.micinf.2012.05.011 22634347

[4] Koch-Nolte F Kernstock S Mueller-Dieckmann C Weiss MS Haag F. Mammalian ADP-Ribosyltransferases and ADP-Ribosylhydrolases. Front Biosci (2008) 13:6716–29. 10.2741/3184 18508690

[5] Bazan JF Koch-Nolte F. Sequence and Structural Links Between Distant ADP-Ribosyltransferase Families. Adv Exp Med Biol (1997) 419:99–107. 10.1007/978-1-4419-8632-0_12 9193642

[6] Glowacki G Braren R Firner K Nissen M Kühl M Reche P. The Family of Toxin-Related Ecto-ADP-Ribosyltransferases in Humans and the Mouse. Protein Sci (2009) 11:1657–70. 10.1110/ps.0200602 PMC2373659 12070318

[7] Koch-Nolte F Petersen D Balasubramanian S Haag F Kahlke D Willer T. Mouse T Cell Membrane Proteins Rt6-1 and Rt6-2 are Arginine/Protein Mono(ADPribosyl)transferases and Share Secondary Structure Motifs With ADP-Ribosylating Bacterial Toxins. J Biol Chem (1996) 271:7686–93. 10.1074/jbc.271.13.7686 8631807

[8] Hong S Brass A Seman M Haag F Koch-Nolte F Dubyak GR. Basal and Inducible Expression of the Thiol-Sensitive ART2.1 Ecto-ADP-Ribosyltransferase in Myeloid and Lymphoid Leukocytes. Purinergic Signal (2009) 5:369–83. 10.1007/s11302-009-9162-2 PMC2717319 19404775

[9] Koch-Nolte F Duffy T Nissen M Kahl S Killeen N Ablamunits V. A New Monoclonal Antibody Detects a Developmentally Regulated Mouse Ecto-ADP-Ribosyltransferase on T Cells: Subset Distribution, Inbred Strain Variation, and Modulation Upon T Cell Activation. J Immunol (1999) 163:6014–22. 10570289

[10] Adriouch S Bannas P Schwarz N Fliegert R Guse AH Seman M. ADP-Ribosylation at R125 Gates the P2X7 Ion Channel by Presenting a Covalent Ligand to its Nucleotide Binding Site. FASEB J (2008) 22:861–9. 10.1096/fj.07-9294com 17928361

[11] Rissiek B Haag F Boyer O Koch-Nolte F Adriouch S. P2X7 on Mouse T Cells: One Channel, Many Functions. Front Immunol (2015) 6:204. 10.3389/fimmu.2015.00204 26042119 PMC4436801

[12] Seman M Adriouch S Scheuplein F Krebs C Freese D Glowacki G. NAD-Induced T Cell Death: ADP-Ribosylation of Cell Surface Proteins by ART2 Activates the Cytolytic P2X7 Purinoceptor. Immunity (2003) 19:571–82. 10.1016/s1074-7613(03)00266-8 14563321

[13] Scheuplein F Schwarz N Adriouch S Krebs C Bannas P Rissiek B. NAD+ and ATP Released From Injured Cells Induce P2X7-Dependent Shedding of CD62L and Externalization of Phosphatidylserine by Murine T Cells. J Immunol (2009) 182:2898–908. 10.4049/jimmunol.0801711 19234185

[14] Rissiek B Danquah W Haag F Koch-Nolte F. Technical Advance: A New Cell Preparation Strategy That Greatly Improves the Yield of Vital and Functional Tregs and NKT Cells. J Leukocyte Biol (2014) 95:543–9. 10.1189/jlb.0713407 24212099

[15] Stark R Wesselink TH Behr FM Kragten NAM Arens R Koch-Nolte F. TRM Maintenance is Regulated by Tissue Damage *via* P2RX7. Sci Immunol (2018) 3. 10.1126/sciimmunol.aau1022 30552101

[16] Borges da Silva H Wang H Qian LJ Hogquist KA Jameson SC. ARTC2.2/P2RX7 Signaling During Cell Isolation Distorts Function and Quantification of Tissue-Resident CD8^+^ T Cell and Invariant NKT Subsets. JI (2019) 202:2153–63. 10.4049/jimmunol.1801613 PMC6424602 30777922

[17] Georgiev H Ravens I Papadogianni G Malissen B Förster R Bernhardt G. Blocking the ART2.2/P2X7-System Is Essential to Avoid a Detrimental Bias in Functional CD4 T Cell Studies. Eur J Immunol (2018) 48:1078–81. 10.1002/eji.201747420 29508376

[18] Rissiek B Lukowiak M Raczkowski F Magnus T Mittrücker H-W Koch-Nolte F. *In Vivo* Blockade of Murine ARTC2.2 During Cell Preparation Preserves the Vitality and Function of Liver Tissue-Resident Memory T Cells. Front Immunol (2018) 9:1580. 10.3389/fimmu.2018.01580 30038627 PMC6046629

[19] Muyldermans S. Nanobodies: Natural Single-Domain Antibodies. Annu Rev Biochem (2013) 82:775–97. 10.1146/annurev-biochem-063011-092449 23495938

[20] Wesolowski J Alzogaray V Reyelt J Unger M Juarez K Urrutia M. Single Domain Antibodies: Promising Experimental and Therapeutic Tools in Infection and Immunity. Med Microbiol Immunol (2009) 198:157–74. 10.1007/s00430-009-0116-7 PMC2714450 19529959

[21] Ingram JR Schmidt FI Ploegh HL. Exploiting Nanobodies’ Singular Traits. Annu Rev Immunol (2018) 36:695–715. 10.1146/annurev-immunol-042617-053327 29490163

[22] De Genst E Silence K Decanniere K Conrath K Loris R Kinne J. Molecular Basis for the Preferential Cleft Recognition by Dromedary Heavy-Chain Antibodies. Proc Natl Acad Sci (2006) 103:4586–91. 10.1073/pnas.0505379103 PMC1450215 16537393

[23] Koch-Nolte F Reyelt J Schößow B Schwarz N Scheuplein F Rothenburg S. Single Domain Antibodies From Llama Effectively and Specifically Block T Cell Ecto-ADP-Ribosyltransferase ART2.2 *In Vivo*. FASEB J (2007) 21:3490–8. 10.1096/fj.07-8661com 17575259

[24] Hubert S Rissiek B Klages K Huehn J Sparwasser T Haag F. Extracellular NAD+ Shapes the Foxp3+ Regulatory T Cell Compartment Through the ART2–P2X7 Pathway. J Exp Med (2010) 207:2561–8. 10.1084/jem.20091154 PMC2989765 20975043

[25] Danquah W Meyer-Schwesinger C Rissiek B Pinto C Serracant-Prat A Amadi M. Nanobodies That Block Gating of the P2X7 Ion Channel Ameliorate Inflammation. Sci Trans Med (2016) 8:366ra162. 10.1126/scitranslmed.aaf8463 27881823

[26] Koch-Nolte F Eichhoff A Pinto-Espinoza C Schwarz N Schäfer T Menzel S. Novel Biologics Targeting the P2X7 Ion Channel. Curr Opin Pharmacol (2019) 47:110–8. 10.1016/j.coph.2019.03.001 30986625

[27] Kaczmarek-Hajek K Zhang J Kopp R Grosche A Rissiek B Saul A. Re-Evaluation of Neuronal P2X7 Expression Using Novel Mouse Models and a P2X7-Specific Nanobody. eLife (2018) 7:e36217. 10.7554/eLife.36217 30074479 PMC6140716

[28] Saunders KO. Conceptual Approaches to Modulating Antibody Effector Functions and Circulation Half-Life. Front Immunol (2019) 10:1296. 10.3389/fimmu.2019.01296 31231397 PMC6568213

[29] Tijink BM Laeremans T Budde M Stigter-van Walsum M Dreier T de Haard HJ. Improved Tumor Targeting of Anti-Epidermal Growth Factor Receptor Nanobodies Through Albumin Binding: Taking Advantage of Modular Nanobody Technology. Mol Cancer Ther (2008) 7:2288–97. 10.1158/1535-7163.MCT-07-2384 18723476

[30] Ghetie V Popov S Borvak J Radu C Matesoi D Medesan C. Increasing the Serum Persistence of an IgG Fragment by Random Mutagenesis. Nat Biotechnol (1997) 15:637–40. 10.1038/nbt0797-637 9219265

[31] Chen H. Manufacturing of Adeno-Associated Viruses, for Example: AAV2. Methods Mol Biol (2011) 737:235–46. 10.1007/978-1-61779-095-9_10 21590400

[32] Balazs AB Chen J Hong CM Rao DS Yang L Baltimore D. Antibody-Based Protection Against HIV Infection by Vectored Immunoprophylaxis. Nature (2011) 481:81–4. 10.1038/nature10660 PMC3253190 22139420

[33] Nimmerjahn F Ravetch JV. Divergent Immunoglobulin G Subclass Activity Through Selective Fc Receptor Binding. Science (2005) 310:1510–2. 10.1126/science.1118948 16322460

[34] Anderson KG Mayer-Barber K Sung H Beura L James BR Taylor JJ. Intravascular Staining for Discrimination of Vascular and Tissue Leukocytes. Nat Protoc (2014) 9:209–22. 10.1038/nprot.2014.005 PMC4428344 24385150

[35] Schenkel JM Masopust D. Tissue-Resident Memory T Cells. Immunity (2014) 41:886–97. 10.1016/j.immuni.2014.12.007 PMC4276131 25526304

[36] Mueller SN Mackay LK. Tissue-Resident Memory T Cells: Local Specialists in Immune Defence. Nat Rev Immunol (2016) 16:79–89. 10.1038/nri.2015.3 26688350

[37] Jacobson KA Müller CE. Medicinal Chemistry of Adenosine, P2Y and P2X Receptors. Neuropharmacology (2016) 104:31–49. 10.1016/j.neuropharm.2015.12.001 26686393 PMC4871727

[38] Burnstock G Knight GE. The Potential of P2X7 Receptors as a Therapeutic Target, Including Inflammation and Tumour Progression. Purinergic Signal (2018) 14:1–18. 10.1007/s11302-017-9593-0 PMC5842154 29164451

[39] Sluyter R. The P2X7 Receptor. Adv Exp Med Biol (2017) 1051:17–53. 10.1007/5584_2017_59 28676924

[40] De Marchi E Orioli E Dal Ben D Adinolfi E. P2X7 Receptor as a Therapeutic Target. Adv Protein Chem Struct Biol (2016) 104:39–79. 10.1016/bs.apcsb.2015.11.004 27038372

[41] Di Virgilio F Sarti AC Falzoni S De Marchi E Adinolfi E. Extracellular ATP and P2 Purinergic Signalling in the Tumour Microenvironment. Nat Rev Cancer (2018) 18:601–18. 10.1038/s41568-018-0037-0 30006588

[42] De Marchi E Orioli E Pegoraro A Sangaletti S Portararo P Curti A. The P2X7 Receptor Modulates Immune Cells Infiltration, Ectonucleotidases Expression and Extracellular ATP Levels in the Tumor Microenvironment. Oncogene (2019) 38:3636–50. 10.1038/s41388-019-0684-y PMC6756114 30655604

[43] Adinolfi E Raffaghello L Giuliani AL Cavazzini L Capece M Chiozzi P. Expression of P2X7 Receptor Increases *In Vivo* Tumor Growth. Cancer Res (2012) 72:2957–69. 10.1158/0008-5472.CAN-11-1947 22505653

[44] Demeules M Scarpitta A Abad C Gondé H Hardet R Pinto-Espinoza C. Evaluation of P2X7 Receptor Function in Tumor Contexts Using rAAV Vector and Nanobodies (AAVnano). Front Oncol (2020) 10:1699. 10.3389/fonc.2020.01699 33042812 PMC7518291

[45] Bartlett R Stokes L Sluyter R. The P2X7 Receptor Channel: Recent Developments and the Use of P2X7 Antagonists in Models of Disease. Pharmacol Rev (2014) 66:638–75. 10.1124/pr.113.008003 24928329

[46] Di Virgilio F Giuliani AL Vultaggio-Poma V Falzoni S Sarti AC. Non-Nucleotide Agonists Triggering P2X7 Receptor Activation and Pore Formation. Front Pharmacol (2018) 9:39. 10.3389/fphar.2018.00039 29449813 PMC5799242

[47] Stokes L Bidula S Bibič L Allum E. To Inhibit or Enhance? Is There a Benefit to Positive Allosteric Modulation of P2X Receptors? Front Pharmacol (2020) 11:627. 10.3389/fphar.2020.00627 32477120 PMC7235284

[48] Di Virgilio F Dal Ben D Sarti AC Giuliani AL Falzoni S. The P2X7 Receptor in Infection and Inflammation. Immunity (2017) 47:15–31. 10.1016/j.immuni.2017.06.020 28723547

[49] Douguet L Janho Dit Hreich S Benzaquen J Seguin L Juhel T Dezitter X. A Small-Molecule P2RX7 Activator Promotes Anti-Tumor Immune Responses and Sensitizes Lung Tumor to Immunotherapy. Nat Commun (2021) 12:653. 10.1038/s41467-021-20912-2 33510147 PMC7843983

