# Supplementary material for: A Methodological Approach Using rAAV Vectors Encoding Nanobody-Based Biologics to Evaluate ARTC2.2 and P2X7 In Vivo
Source: Front Immunol. 2021 Aug 19;12:704408. doi: 10.3389/fimmu.2021.704408 (PMC8417108; doi:10.3389/fimmu.2021.704408)
Supplement: Supplementary file 1 [file DataSheet_1.pdf]

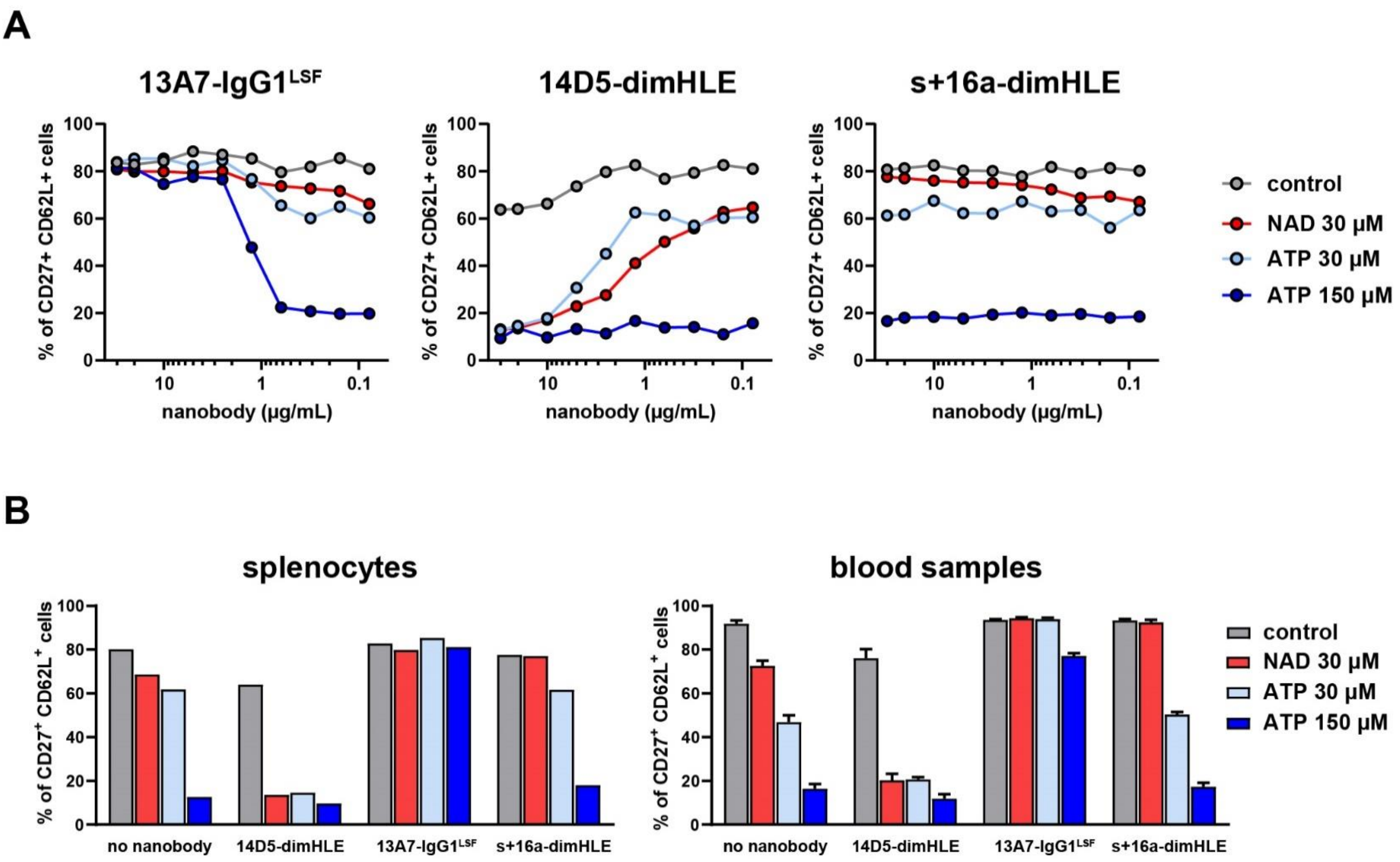

Figure S1: Recombinant nanobody constructs modulate P2X7-dependent shedding of CD27 and CD62L *in vitro*

(A) Splenocytes for untransduced mice were collected and incubated for 60 min à 4°C without recombinant nanobodies (control) or with decreasing concentrations of the indicated recombinant 13A7-IgG1^{LSF}, 14D5-dimHLE or s+16a-dimHLE nanobody-constructs. Cells were then incubated for 15 min at 37°C after addition of PBS (grey), 30 µM NAD (red), 30 µM ATP (cyan) or 150 µM ATP (blue). Dose-responses curves indicate the percentages of CD27$^+$CD62L$^+$ cells among the gated CD4$^+$CD25$^-$ T cells. **(B)** Splenocytes (left) or pooled blood samples (right) from untransduced C57BL/6 mice were collected and incubated for 60 min at 4°C with or without the indicated recombinant nanobody-construct 13A7- IgG1^{LSF}, 14D5-dimHLE or s+16a-dimHLE added at a saturating concentration of 20 µg/ml. Cells were then incubated for 15 min at 37°C after addition of PBS (grey), 30 µM NAD$^+$ (red), 30 µM ATP (cyan) or 150 µM ATP (blue). Bar graphs represent mean percentages of CD27$^+$CD62L$^+$ cells among the gated CD4$^+$CD25$^-$ T cells and errors bars represent SEM, (n=1 for splenocytes and n=3 for blood samples).

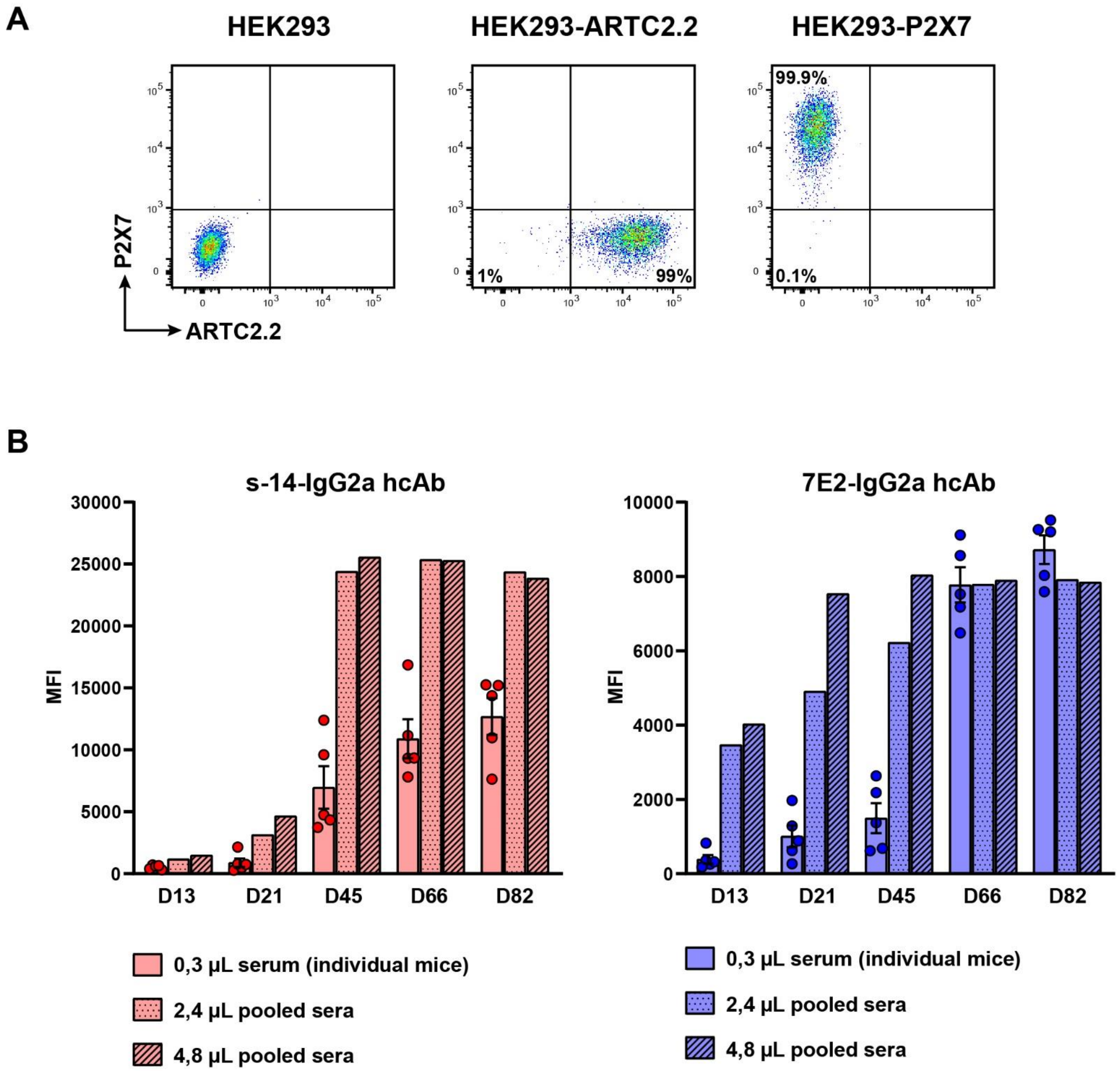

Figure S2: Kinetic and relative abundance in the circulation of the nanobody constructs produced *in vivo* following a single injection of the corresponding AAVnano

(A) HEK-293 cells were transduced by a retroviral vector to express ARTC2.2 (HEK293-ARTC2.2) or P2X7 (HEK293-P2X7), these cells were further used here to analyze the relative abundance of 7E2-IgG2a hcAb or s-14-IgG2a hcAb in the sera or plasma of transduced mice overtime. **(B)** C57BL/6 mice were injected with PBS or with rAAV8 encoding 7E2-IgG2a or s-14-IgG2a hcAb. Plasma or sera were collected on days 13, 21, 45,

66 and 82 post rAAV8 i.m. injection. To monitor the kinetic and to estimate the relative abundance of hcAb production in the circulation, HEK293-ARTC2.2 cells (for detection of s-14-IgG2a hcAb, in red) or HEK293-P2X7 cells (for detection of 7E2-IgG2a, in blue) were incubated with 0.3 µL of serum collected from each individual mouse or with the corresponding quantity of plasma. In addition, cells were also incubated with higher quantities of pooled sera (or corresponding quantity of plasma) to estimate the level of saturation at each time point of the kinetic. For that, sera (or equivalent quantities of plasma) collected from individual mouse at each time point were pooled, and 2.4 µL (doted pattern), or 4.8 µL (hatched pattern) were used to stain the cells. After washing, bound hcAbs were detected using a biotinylated IgG2a-specific secondary antibody followed with streptavidin-PE. Closed circles correspond to the mean fluorescence intensity (MFI) staining obtained from the sera (or equivalent quantities of plasma) of each individual mouse (n=5). Bar graphs and errors bars represent respectively the mean and SEM values at each analyzed time points. For negative control, sera (or corresponding quantity of plasma) collected from untransduced mice were pooled and used. Resulting background levels were estimated to correspond to a MFI of 243 ± 8 for detection of s-14-IgG2a hcAb (left histograms) and to a MFI of 168 ± 23 for detection of 7E2-IgG2a hcAb (blue histograms).

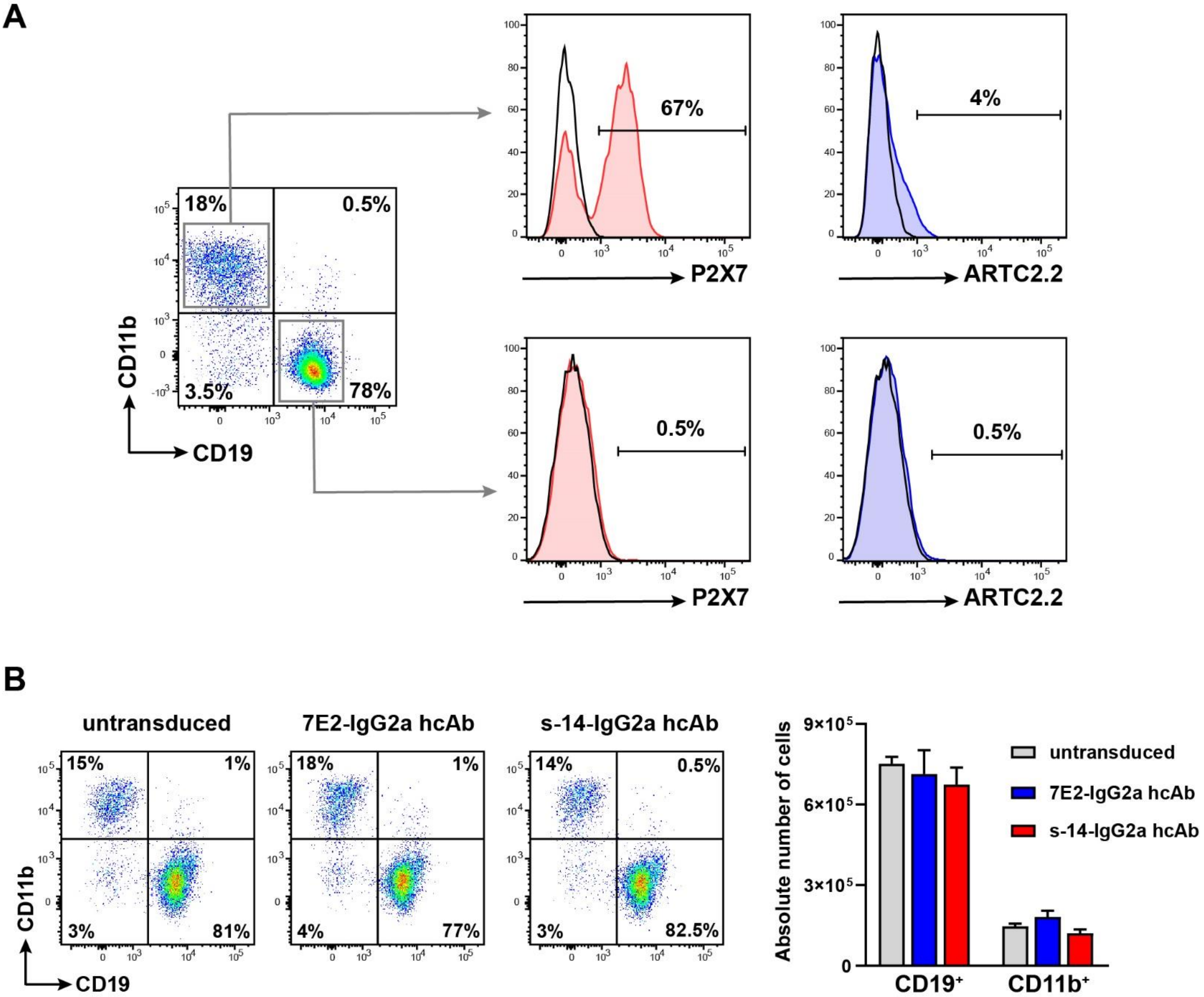

Figure S3: Blood CD19$^+$ B cells and myeloid non-granulocytic CD11b$^+$ cells are unaffected by nanobody-based IgG2a hcAb constructs

(A) Flow cytometry analyses illustrating the level of expression of P2X7 and ARTC2.2 on the surface of blood CD19$^+$ B cells and on non-granulocytic CD11b$^+$ cells of untreated mice. Cells were gated on CD45$^+$CD4$^-$CD8$^-$ cells, and dead cells and granulocytes were excluded based on the scatters. Flow cytometry histograms represent staining of P2X7 (red) or ARTC2.2 (blue) or negative controls (black line) corresponding to isotype control (for ARTC2.2 staining), or to secondary antibody alone (for P2X7 staining). Numbers correspond to the percentage of cells in each indicated gate. Staining was performed using fluorochrome

conjugated antibodies specific to CD45 (coupled to BV510), CD11b (FITC), CD19 (PerCP-Cy5.5), or ARTC2.2 (AF647). Staining for P2X7 was performed using polyclonal rabbit antibody K1G followed by a polyclonal secondary anti-rabbit IgG coupled to APC. **(B)** C57BL/6 mice were injected with PBS (untransduced, grey) or with rAAV8 encoding 7E2-IgG2a (blue) or s-14-IgG2a (red) hcAb. On day 82, blood samples were collected and stained using fluorochrome conjugated antibodies specific to CD45 (coupled to BV510), CD11b (FITC), CD19 (PerCP-Cy5.5). Representative flow cytometry dot plots were gated on $CD45^{+}CD4^{-}CD8^{-}$ cells, and dead cells and granulocytes were excluded based on the scatters. Numbers correspond to the percentage of cells in each indicated quadrant. Histogram graphs represent the absolute numbers of $CD11b^{-}CD19^{+}$ B cells ($CD19^{+}$) and the absolute numbers of myeloid non-granulocytic $CD11b^{+}CD19^{-}$ cells ($CD11b^{+}$) in each indicated group of mice. Errors bars represent the SEM, n=5.
